# Trait-Based Comparison of Coral and Sponge Microbiomes

**DOI:** 10.1038/s41598-020-59320-9

**Published:** 2020-02-11

**Authors:** Cara L. Fiore, Jessica K. Jarett, Georg Steinert, Michael P. Lesser

**Affiliations:** 1 University of New Hampshire, Department of Molecular, Cellular and Biomedical Sciences, School of Marine Science and Ocean Engineering, Durham, NH USA; 2 Present Address: Appalachian State University, Biology Department, Boone, NC USA; 3 Present Address: AnimalBiome, Oakland, CA USA; 4 Institute for Chemistry and Biology of the Marine Environment, Carl-von-Ossietzky University Oldenburg, Wilhelmshaven, Germany; 5 Present Address: GEOMAR Helmholtz Centre for Ocean Research Kiel, Marine Symbioses, Kiel, Germany

**Keywords:** Environmental microbiology, Symbiosis

## Abstract

Corals and sponges harbor diverse microbial communities that are integral to the functioning of the host. While the taxonomic diversity of their microbiomes has been well-established for corals and sponges, their functional roles are less well-understood. It is unclear if the similarities of symbiosis in an invertebrate host would result in functionally similar microbiomes, or if differences in host phylogeny and environmentally driven microhabitats within each host would shape functionally distinct communities. Here we addressed this question, using metatranscriptomic and 16S rRNA gene profiling techniques to compare the microbiomes of two host organisms from different phyla. Our results indicate functional similarity in carbon, nitrogen, and sulfur assimilation, and aerobic nitrogen cycling. Additionally, there were few statistical differences in pathway coverage or abundance between the two hosts. For example, we observed higher coverage of phosphonate and siderophore metabolic pathways in the star coral, *Montastraea cavernosa*, while there was higher coverage of chloroalkane metabolism in the giant barrel sponge, *Xestospongia muta*. Higher abundance of genes associated with carbon fixation pathways was also observed in *M. cavernosa*, while in *X. muta* there was higher abundance of fatty acid metabolic pathways. Metagenomic predictions based on 16S rRNA gene profiling analysis were similar, and there was high correlation between the metatranscriptome and metagenome predictions for both hosts. Our results highlight several metabolic pathways that exhibit functional similarity in these coral and sponge microbiomes despite the taxonomic differences between the two microbiomes, as well as potential specialization of some microbially based metabolism within each host.

## Introduction

Microbial symbionts have played a critical role in shaping the evolution of multicellular life by facilitating and promoting host defense^1^, nutrient acquisition^2^, and species diversification^3^. For example, in coral reef ecosystems, intracellular dinoflagellates in the Family Symbiodiniaceae occur as the primary mutualistic symbionts within the coral host and are essential to the survival and growth of the coral host, while receiving a suitable habitat and essential nutrients in return^4,5^. Prokaryotic symbionts also contribute to the success of coral reef organisms, including corals and sponges^6–9^, by providing bioactive compounds for chemical defenses^10,11^, recycling host waste^12,13^, and in some cases providing a reliable source of vitamins and nutrients^14–17^. Numerous studies have characterized the taxonomic composition of microbes in corals and sponges^18,19^; however, our understanding of the multiple functional roles of these symbionts is still incomplete.

Corals and sponges harbor remarkably diverse prokaryotic symbionts with thousands of unique taxa in many coral and sponge species^20,21^. Many of these taxa persist across different host species (i.e., the core microbiome) over broad spatial scales^9,22–24^, and in some cases these host-associated microbial communities are more stable over time than free-living communities^24–26^, but see Sweet *et al*.^27^ for another viewpoint. Recent work, however, has demonstrated a more pervasive environmental component to the microbiome composition of coral reef host organisms, with a high degree of overlap for environmental microbial taxa in sponges specifically^22^ although high specificity of prokaryotic taxa in sponges is also well documented^19,22,28^. Sponges contain the highest number of unique taxa relative to other benthic host organisms, highlighting gaps in our knowledge in regards to microbiome structure and function within sponges and other coral reef organisms^22^. In both corals and sponges, the overall taxonomic composition of microbial consortia is influenced by host phylogeny^19,27,28^, other symbionts (e.g., Symbiodiniaceae) and environmental factors^19,27–31^, underscoring the multiple, complex influences on microbiome structure (reviewed for corals^7,9,18^; sponges^31,32^).

Recent metagenomic and metatranscriptomic studies on corals and sponges have revealed a complex picture of microbial functions. The cycling of essential nutrients, primarily carbon (C), nitrogen (N), sulfur (S), and phosphorus (P) (see below; reviewed in^8,18,31,32^) has been the best studied to date. The fixation of carbon in the form of CO_2_ by symbionts and subsequent translocation of photosynthate to the host is well documented for Symbiodiniaceae in corals but has also been observed for *Cyanobacteria* and Symbiodiniaceae in several sponges^16,33,34^. The discovery of multiple carbon fixation pathways in coral- and sponge-associated microbes, including the Wood-Ljungdahl pathway, reverse tricarboxylic acid (TCA) cycle, and reductive acetyl-CoA pathway, indicate a far more complex system for carbon cycling and potential host-microbe interactions^17,35–38^ than previously believed. Furthermore, microbes in corals and sponges can transform N^14,15^, S^39,40^, P^41,42^, and diverse organic compounds in dissolved organic matter^43–47^ into microbial biomass that can be consumed by the host or into compounds that provide direct or indirect benefits to the host (e.g., ammonia from fixed N_2_). It is important to note, however, that microbial and host physiologies have not necessarily evolved towards beneficial interactions as a means of coexistence, even when translocation of metabolic products is considered^14,16,17,48^. Arguably, it is more typical that each member is selected upon to survive in a complex system, often utilizing by-products of metabolism from other members (e.g., syntrophy) in an ecosystem that is the holobiont^49^.

Different structural characteristics of corals and sponges create different microhabitats that influence the composition of their microbiomes. Both host organisms have an open system where microbes are exposed to environmental conditions in the surrounding seawater. Sponges contain multiple cell types that form different layers, as well as the aquiferous system, which includes choanocyte chambers for filtering water. Variability in the flow rate of water through the sponge body creates microhabitats based on oxygen gradients^12^, facilitating physiologically diverse microbial communities. Scleractinian corals contain an outer mucus layer, and tissue layers with and without algal symbionts, as well as a calcium carbonate skeleton, each of which forms a unique microhabitat^7,50,51^. Corals also exhibit a strong diurnal oxygen gradient, with hyperoxia during the day from algal photosynthesis, and hypoxia at night^52^, creating temporally-mediated niches for diverse microbial metabolic processes, such as nitrogen fixation^8,14^. The different characteristics of each host species and their co-occurrence in the same habitat provide an opportunity to examine both specialized and convergent aspects of their microbiomes. Similar comparisons have been made within phylogenetically diverse sponge microbiomes, which indicated functional convergence in five main areas: (a) nitrogen metabolism, (b) nutrient utilization and nutritional interactions with the host, (c) resistance to environmental and host-specific stress, (d) eukaryotic-like proteins involved in host-microbe interaction, and (e) horizontal gene transfer^53^. However, there are few studies that have compared functional diversity and potential convergence in microbiomes between invertebrate phyla^22,54^.

**Figure 1:**
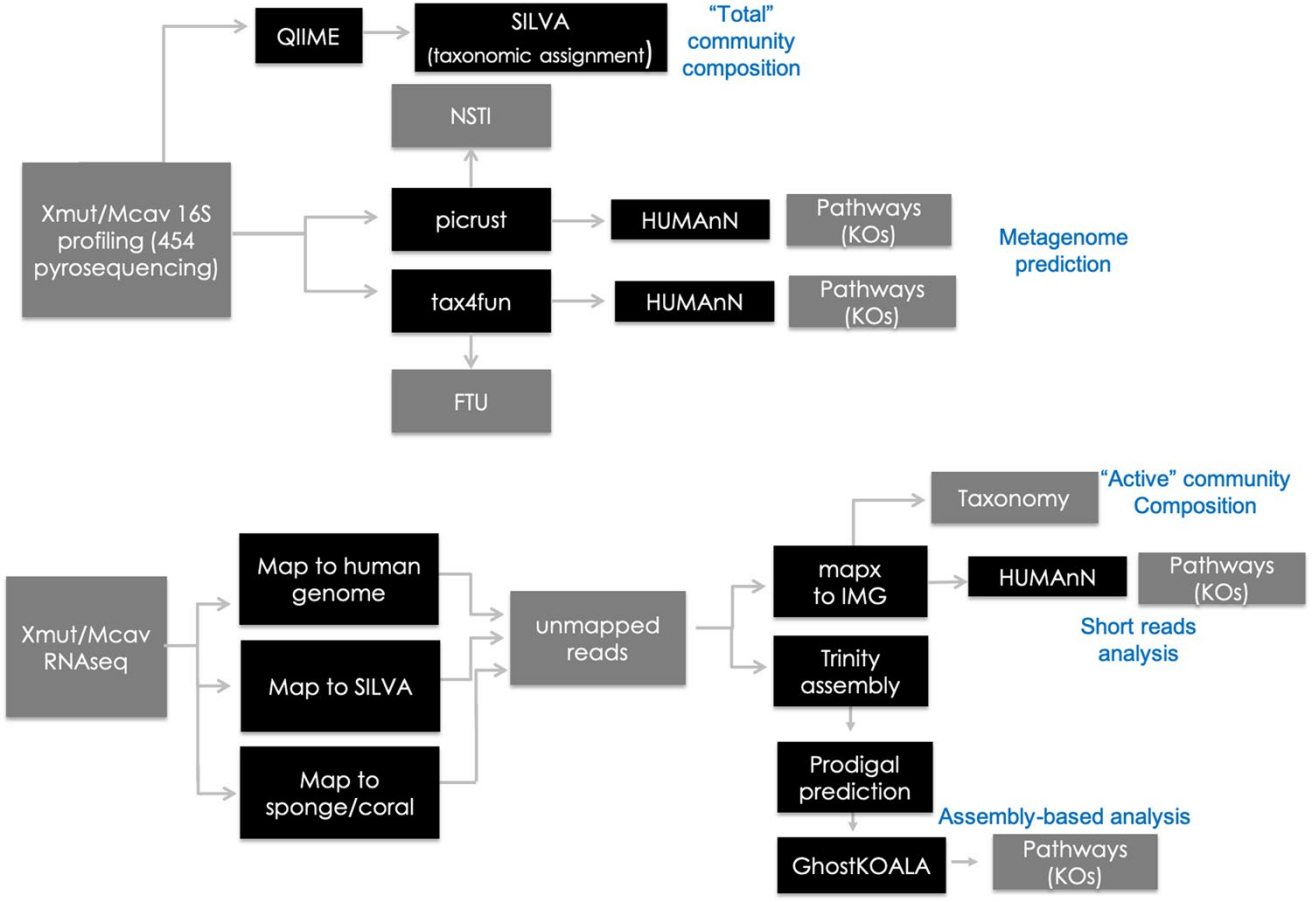
Overview of metatranscriptome and microbiome profiling methods. Gray boxes represent data types while black boxes represent a data analysis program and/or process.

Here, we integrate previously published^17,55^ RNAseq data from the microbiome of the great star coral, *Montastraea cavernosa*, and the giant barrel sponge, *Xestospongia muta*, along with new analyses of these metatranscriptomes, to advance our understanding of microbiome function in these two abundant and phylogenetically distant host species in the Caribbean basin. The analysis presented here includes a unique approach that directly analyzes quality-trimmed short-reads from RNA sequencing. This short-read analysis is paired with an assembly of each metatranscriptome to allow for more in-depth analysis of metabolic pathways (Fig. 1). While advances in computation now make *de novo* metatranscriptome assembly a more feasible approach than it used to be, the complementary approaches provide novel insight into the functional repertoire of two distinct host species. Furthermore, while there is a strong interest in environmental microbiome analysis^22,56^, there no published studies (to our knowledge) that compare metatranscriptome profiles across host phyla. The scleractinian coral, *M. cavernosa* exists in two co-occurring morphs, orange and brown; the orange morph contains more abundant cyanobacteria and is capable of much higher rates of nitrogen fixation than the brown morph^14,55^. *Xestospongia muta* is an abundant sponge that can grow to massive sizes on many Caribbean reefs, with important implications for nutrient dynamics in those habitats^57–59^. Finally, we compared prokaryotic community predictions from 16S rRNA gene amplicon sequences^55,60^ to functional activity to assess the accuracy of predictive approaches (Fig. 1). While this study is limited to the analysis of one coral species and one sponge species, the comparison of the two hosts sheds light on core processes related to symbiosis.

## Results and Discussion

### Comparison of total and active microbial communities

**Figure 2:**
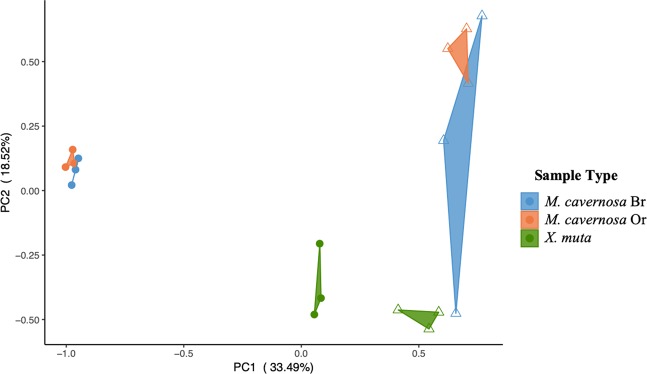
Principal components analysis of active and total microbial communities in the coral *Montastraea cavernosa* (brown (Br) and orange (Or) color morphs) and sponge, *Xestospongia muta*. Open triangles represent total microbial communities (16S rRNA genes) and closed circles represent active communities (mRNA). Total microbial communities were identified by 16S rRNA gene profiling performed previously (Fiore *et al*.^60^; Jarett *et al*.^55^). Active microbial communities were identified by querying prokaryotic genomes in the Integrated Microbial Genomes (IMG) database (see Methods).

The total microbial community, as measured by 16S rRNA amplicon sequencing in previous work^55,60^ differs between *M. cavernosa* and *X. muta* hosts (Supplementary Fig. S1). Data from the current study shows that the target host species also differed in their active communities under the environmental conditions when collections occurred, determined here by mapping mRNA reads to a reference database to assign taxonomy (Fig. 2, Supplementary Information S1, Supplementary Information S2). Notably, in *M. cavernosa* and *X. muta*, *Cyanobacteria*, *Proteobacteria*, and *Firmicutes* were more transcriptionally active than would be predicted by the abundance of 16S rRNA genes (Supplementary Fig. S1; Supplementary Information S2). These major groups may underlie functional characteristics in the host microbiomes described below. Meanwhile, the largest proportion of the active community in *M. cavernosa* included *Beta*- and *Gammaproteobacteria* and *Bacteroidetes* and in *X. muta* included *Cyanobacteria*, *Beta*- and *Alphaproteobacteria*. The differences highlighted between the total and active groups within each host and between host communities and may drive some of the difference visualized by principal components analysis (PCA) (Fig. 2).

The taxonomic differences highlighted here between the *M. cavernosa* and *X. muta* microbiomes used in this study could imply functional differences in activity and potentially differences in niche partitioning within the holobiont^61,62^. Alternatively, similar functional traits may dominate metabolic activity in the two different host taxa, as has been described in other studies^53,63–65^. We address this question below by comparing the functional profile of *M. cavernosa* and *X. muta* using metatranscriptomics. We note however, that samples of coral and sponge were collected at the same depth (15 m) but at different times of day (morning and midday, respectively) and in different locations in the Caribbean. These temporal and environmental factors could influence differences in both the composition of the active community profile (identity) and the functional profile (transcripts) between the two host organisms.

### Functional overview of the sponge and coral microbiome

Profiles of functional gene expression were generated with two approaches; the first provided a functional profile of metabolic pathways (short-read analysis) and the second provided more in-depth analysis of specific metabolic pathways (assembly analysis) (Fig. 1). The short-read analysis used the mapx algorithm to map RNA reads to the IMG prokaryotic genomes followed by analysis with HUMAnN^66^ to produce estimates of coverage and abundance for KEGG^67^ pathways (see Methods). These results were subsequently analyzed in STAMP^68^ by PCA and analysis of variance (ANOVA) with an effect size threshold of 0.8 and Benjamin-Hochberg FDR correction for *p*-values^69^ (see Supplementary Information S1). Meanwhile, assembly of the RNA reads into putative mRNA transcripts (i.e., contigs) allowed us to map and visualize gene expression for specific KEGG pathways of interest. Hereafter, we refer to read coverage or abundance (from the short-read analysis) and transcript presence (assembly analysis) in a metabolic pathway. Reads per sample varied across samples but were not significantly different between coral, *M. cavernosa* (average 40,832,418 (±3,815,419)^55^), and sponge, *X. muta* (42,230,246 (±9,246,767)^17^) (*t*-test, *p* = 0.01). We note, however, that differences in time of sampling limit the interpretation of our comparisons to some extent.

**Figure 3:**
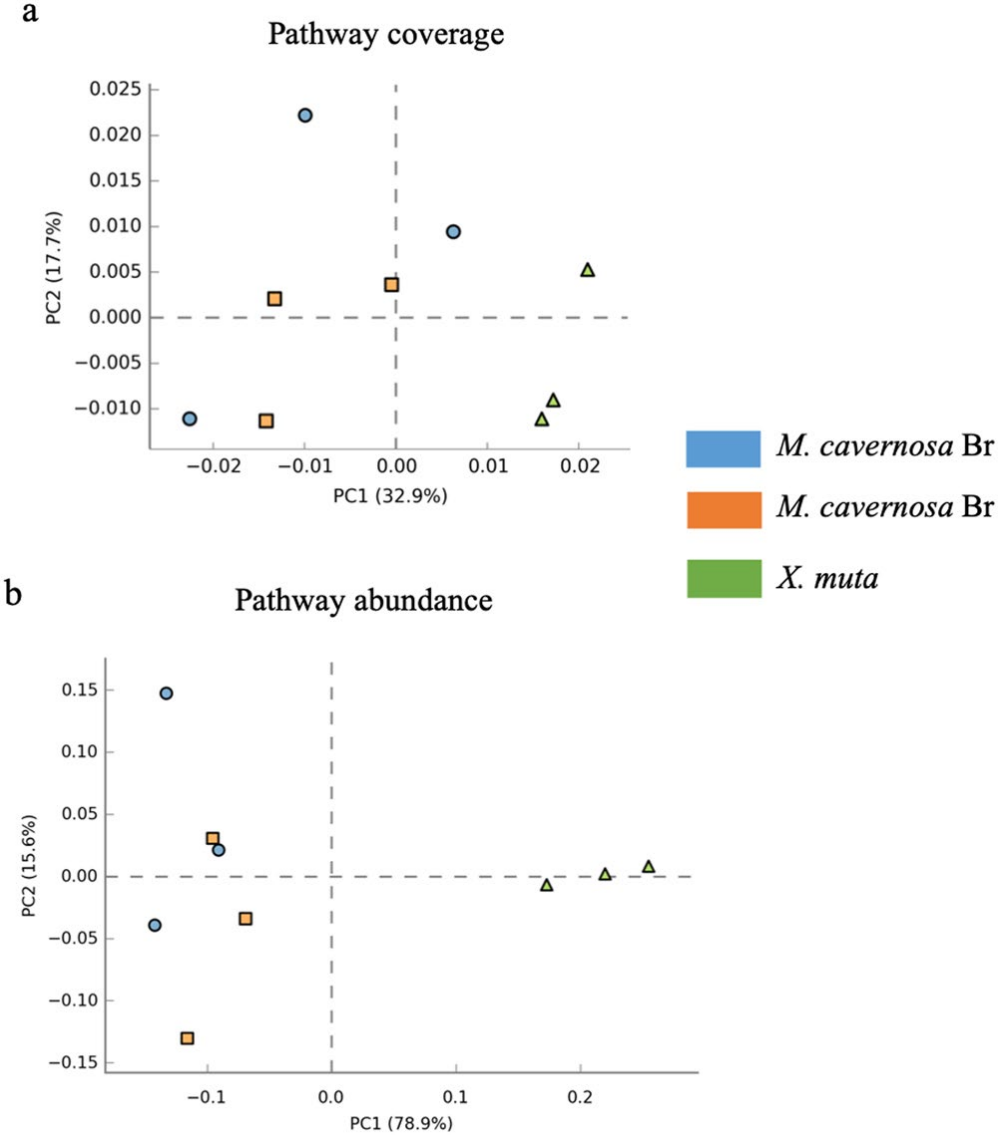
Principal components analysis (PCA) of KEGG Orthology pathways^67^ based on coverage (**a**) and relative abundance (**b**) in the coral, *Montastraea cavernosa* (brown (Br) and orange (Or) color morphs) and sponge, *Xestospongia muta*. KEGG Orthology pathways were assigned, and relative abundance and coverage were calculated, with HUMAnN^66^ based on putative mRNA reads from the prokaryotic community.

In the short-read analysis, we compared the relative coverage and abundance of KOs from symbiont reads in the color morphs of *M. cavernosa* and *X. muta*. There were no differences in pathway abundances between coral color morphs (ANOVA, *p* > 0.05, Fig. S2), so corals were pooled for further analyses. The lack of significant differences within the corals was initially surprising because expression differences were observed in earlier analysis of this dataset^55^; however, the analysis here is based on annotation of short-reads which may highlight an important methodological consideration for future work. The previous work^55^ used assembly analysis and observed some, but few overall, differentially expressed genes between the color morphs. The assembly approach may be more sensitive to differences in transcript abundance. Furthermore, previous work indicated no significant difference in microbial community composition between the color morphs of *M. cavernosa*^55^. When considering all pathways, coral and sponge samples could be separated along by PCA (Fig. 3a), particularly by pathway abundance (Fig. 3b). However, *M. cavernosa* color morphs were always intermingled (Supplementary Fig. S2). These data suggest that the metabolic differences between host species are much larger than those between coral morphs. This difference between species may be largely driven by differences in transcript abundance or differences in the types of pathways they express; each is described below.

**Figure 4:**
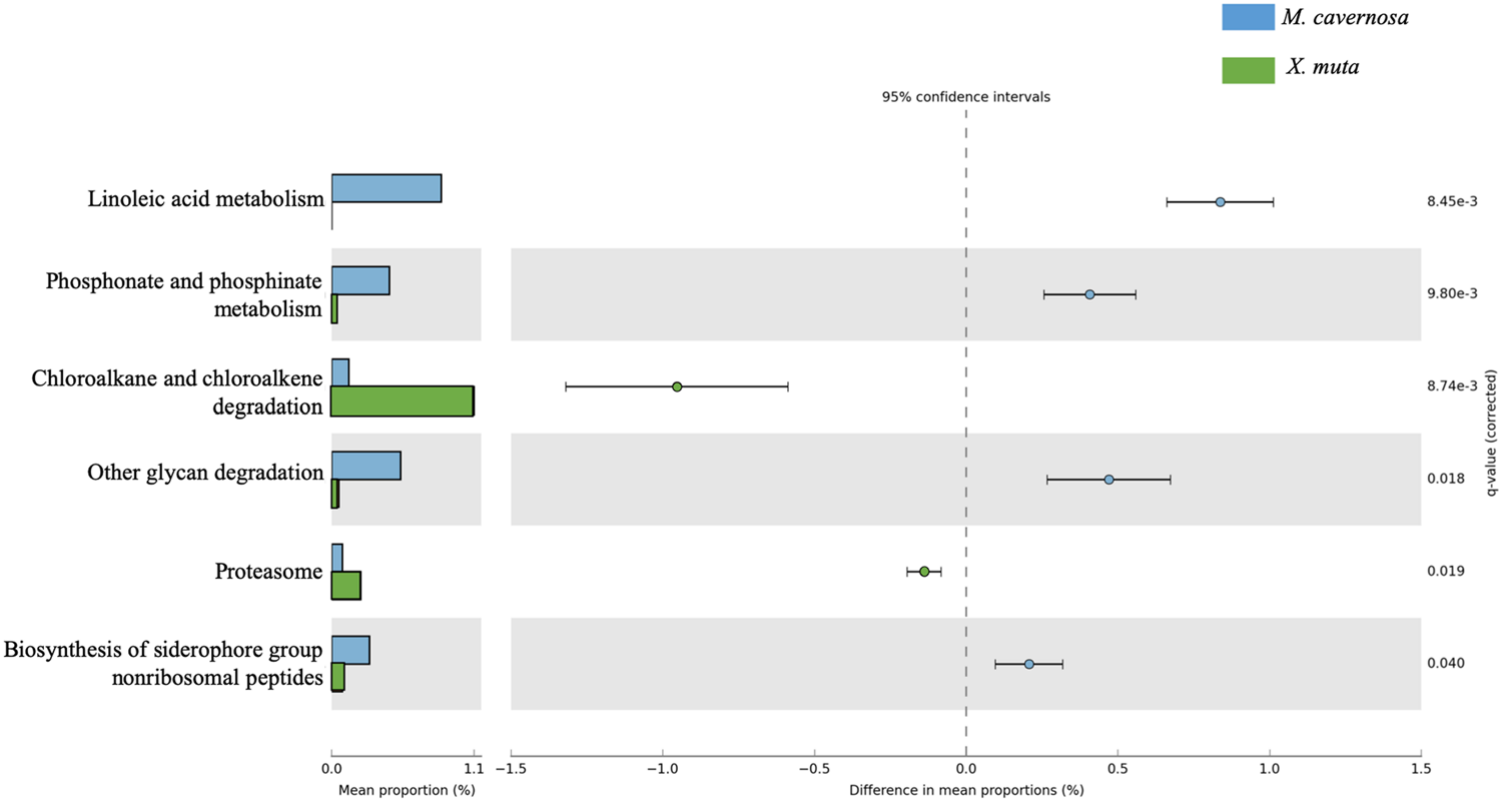
Pair-wise comparisons between KEGG Orthology pathway^67^ coverage in the coral, *Montastraea cavernosa* (n = 6) and sponge, *Xestospongia muta* (n = 3). Difference between groups was assessed with a Welch’s *t*-test with Benjamin-Hochberg FDR correction for q-values and a relative effect size of 0.8. KEGG Orthology pathways were assigned, and relative abundance was calculated, with HUMAnN^66^ based on putative mRNA reads from the prokaryotic community.

Pairwise analysis in STAMP of pathway coverage between *M. cavernosa* and *X. muta*, revealed six pathways that were significantly different in coverage between the pooled *M. cavernosa* and *X. muta* samples (ANOVA, adjusted *p* = 0.003–0.047, Table 1) (Fig. 4), with an additional five pathways below the effect size threshold of 0.8 (Supplementary Fig. S3). Linoleic acid metabolism, phosphonate and phosphinate metabolism, other glycan degradation, and biosynthesis of siderophore group nonribosomal peptide pathways had significantly higher coverage in *M. cavernosa*, while chloroalkane and chloroalkene degradation and proteasome pathways were significantly higher in coverage in *X. muta* (Fig. 4).

**Table 1:** Summary of pair-wise comparisons of pathway coverage and abundance between the coral, *Montastraea cavernosa* (Mcav) and the sponge, *Xestospongia muta* (Xmuta).

Pathway	*p*-values	*p*-values (corrected)	Mcav: mean rel. freq. (%)	Mcav: std. dev. (%)	Xmuta: mean rel. freq. (%)	Xmuta: std. dev. (%)	Difference between means	95.0% lower CI	95.0% upper CI
***Pathway Coverage***									
Biosynthesis of siderophore group nonribosomal peptides	0.005	0.040	0.290	0.097	0.083	0.001	0.207	0.096	0.319
Chloroalkane and chloroalkene degradation	<0.001	0.009	0.132	0.295	1.085	0.115	−0.953	−1.32	−0.587
Linoleic acid metabolism	<0.001	0.008	0.837	0.153	0.000	0.000	0.837	0.661	1.01
Other glycan degradation	0.001	0.018	0.525	0.146	0.054	0.077	0.471	0.267	0.674
Phosphonate and phosphinate metabolism	<0.001	0.010	0.440	0.123	0.033	0.046	0.408	0.257	0.559
Proteasome	0.001	0.019	0.083	0.049	0.221	0.004	−0.138	−0.194	−0.082
***Pathway Abundance***									
Cell cycle - Caulobacter	0.011	0.047	0.43	0.10	2.26	0.31	−1.83	−2.72	−0.94
Fatty acid biosynthesis	<0.001	0.004	0.76	0.10	1.73	0.09	−0.98	−1.18	−0.77
Lipoic acid metabolism	0.003	0.021	0.22	0.13	1.24	0.16	−1.02	−1.41	−0.63
Oxidative phosphorylation	0.001	0.011	21.13	6.15	3.06	1.06	18.07	11.01	25.14
Photosynthesis	<0.001	0.003	25.60	4.94	1.93	1.91	23.66	17.54	29.78
RNA polymerase	0.003	0.019	0.16	0.06	1.41	0.13	−1.24	−1.60	−0.89

The functional profiles were derived from short-read analysis using IMG prokaryotic genomes and processed with the program HUMAnN^66^ to assign pathway coverage and abundance. Analysis of variance and pair-wise comparisons were performed in the program STAMP^68^ with an effect size threshold 0.8 and Benjamin -Hochberg correction for multiple comparisons.

**Figure 5:**
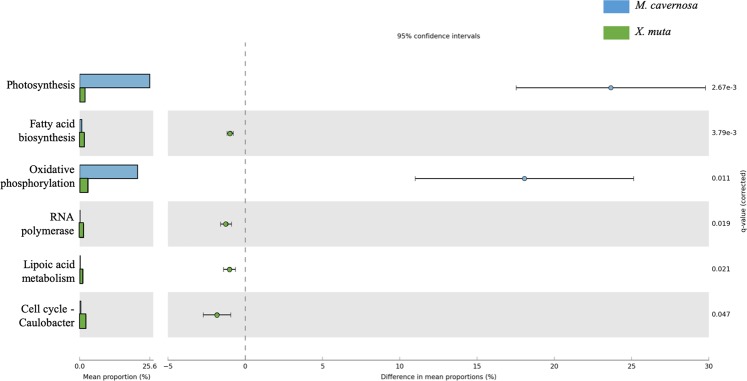
Pair-wise comparisons between KEGG Orthology pathway^67^ abundance in the coral, *Montrastrea cavernosa* (n = 6) and sponge, *Xestospongia muta* (n = 3). Difference between groups was assessed with a Welch’s *t*-test with Benjamin-Hochberg FDR correction for q-values and a relative effect size of 0.8. KEGG Orthology pathways were assigned, and relative abundance was calculated, with HUMAnN^66^ based on putative mRNA reads from the prokaryotic community.

None of the differentially covered pathways were also significantly different in terms of abundance between *M. cavernosa* and *X. muta* (Fig. 5). Six other pathways were significantly different in abundance between *M. cavernosa* and *X. muta* (Table 1, ANOVA, adjusted *p* = 0.008–0.04), with an additional 31 pathways below effect size threshold of 0.8, including lipid metabolism and vitamin B metabolism which have been described as important symbiont pathways in sponges previously (Supplementary Fig. S4). Some metabolic pathways below the effect size threshold for pathway abundance or coverage did overlap between the two host species, such as chloroalkane metabolism (Supplementary Fig. S4). The limited overlap in pathways between the two host organisms that were statistically significant in coverage or abundance, points to a mix of both gene content and gene expression that differ between the *M. cavernosa* and *X. muta* microbiomes.

Lastly, we leveraged the previously published 16S rRNA gene profiling in metagenome prediction to compare the predicted functional profile with the metatranscriptome functional profile. In our study, the HUMAnN-produced KEGG Orthology (KOs) from PICRUSt and Tax4Fun were compared with HUMAnN-produced KOs from metatranscriptome (see Methods). The NSTI values associated with PICRUSt predictions and the FTU values associated with the Tax4Fun predictions were relatively high (Supplementary Table S1). The values of NSTI and FTU provide an estimate of accuracy of the functional prediction based on the taxonomic similarity between the query and reference taxa (see Methods). Here, the high values indicate low similarity between the reference taxa and our samples, and less reliable predictions. Despite the high NSTI and FTU values, there was strong correlation between the functional profiles of the metatranscriptome and metagenome predictions for both coverage and abundance (Table 2). Moreover, there was overlap in the top 25 abundant pathways based on short read analysis of the metatranscriptome and the top 25 pathways predicted based on 16S rRNA gene profiling (Table 3; Supplementary Table S2). Ultimately, the use of multiple approaches (short read analysis, assembly analysis, and metagenome prediction) to compare the functional profiles of *M. cavernosa* and *X. muta* uncovered two key results: (1) similar to other studies, the overlap in functional profiles between the metatranscriptome and the metagenome predictions was relatively high (Table 2), providing support for these metagenome prediction programs in future work, and (2) we observed indications of both convergence and specialization within the host microbiomes.

**Table 2:** Summary of Pearson’s product-moment correlations between predicted KEGG Orthology^67^ pathway coverage or abundance and sequenced metatranscriptomes in the sponge and coral samples.

Prediction	correlation	*t*	*p*-value
**Coverage**			
**Tax4Fun-HUMAnN**			
Xmut	0.82	16.5	<**0.001**
Mcav	0.74	12.9	<**0.001**
**PICRUSt-HUMAnN**			
Xmut	0.82	16.5	<**0.001**
Mcav	0.78	14.3	<**0.001**
**Abundance**			
**Tax4Fun-HUMAnN**			
Xmut	0.36	4.6	<**0.001**
Mcav	0.06	0.74	0.46
**PICRUSt-HUMAnN**			
Xmut	0.49	6.7	<**0.001**
Mcav	0.09	1.04	0.3

KEGG Orthology pathways were assigned, and pathway coverage and abundance were calculated, with HUMAnN^66^ based on mRNA reads or with HUMAnN following PICRUSt^119^ or Tax4Fun^120^ based on 16S rRNA gene profiling. Significant (α = 0.05) *p*-values are in bold.

**Table 3:** Top 25 metabolic pathways of coral and sponge (continued) microbiomes based on short-read analysis.

Pathway	Sponge (*X. muta*)	Coral (*M. cavernosa*)	Unique to *M. cavernosa*
**ko00195: Photosynthesis**	0.019	0.255	ko00830: Retinol metabolism
ko00190: Oxidative phosphorylation	0.030	0.211	ko05200: Pathways in cancer
ko00970: Aminoacyl-tRNA biosynthesis	0.015	0.049	
**ko00450: Selenocompound metabolism**	0.015	0.026	
ko00010: Glycolysis / Gluconeogenesis	0.015	0.023	
ko00750: Vitamin B6 metabolism	0.020	0.018	
**ko00630: Glyoxylate and dicarboxylate metabolism**	0.013	0.018	
**ko00620: Pyruvate metabolism**	0.013	0.018	
**ko00020: Citrate cycle (TCA cycle)**	0.020	0.014	
**ko00710: Carbon fixation in photosynthetic organisms**	0.001	0.013	
ko00270: Cysteine and methionine metabolism	0.011	0.013	
**ko00720: Carbon fixation pathways in prokaryotes**	0.015	0.011	
**ko00290: Valine, leucine and isoleucine biosynthesis**	0.031	0.011	
**ko00660: C5-Branched dibasic acid metabolism**	0.029	0.011	
**ko00030: Pentose phosphate pathway**	0.014	0.011	
ko03010: Ribosome	0.111	0.010	
**ko00250: Alanine, aspartate and glutamate metabolism**	0.013	0.008	
ko00061: Fatty acid biosynthesis	0.017	0.008	
**ko00260: Glycine, serine and threonine metabolism**	0.012	0.007	
ko03018: RNA degradation	0.041	0.007	
**ko00770: Pantothenate and CoA biosynthesis**	0.017	0.007	
**ko00670: One carbon pool by folate**	0.012	0.007	
ko00480: Glutathione metabolism	0.007	0.007	
ko03060: Protein export	0.021	0.006	
ko00910: Nitrogen metabolism	0.005	0.006	
**Pathway**	**Sponge (*X. muta*)**	**Coral (*M. cavernosa*)**	**Unique to** ***X. muta***
ko03010: Ribosome	0.111	0.010	ko05110: Vibrio cholerae infection
ko03018: RNA degradation	0.041	0.007	ko00563: Glycosylphosphatidylinositol(GPI)-anchor biosynthesis
**ko00290: Valine, leucine and isoleucine biosynthesis**	0.031	0.011	ko00642: Ethylbenzene degradation
ko00190: Oxidative phosphorylation	0.030	0.211	ko00909: Sesquiterpenoid biosynthesis
ko00660: C5-Branched dibasic acid metabolism	0.029	0.011	ko00621: Dioxin degradation
**ko04112: Cell cycle - Caulobacter**	0.022	0.004	ko05143: African trypanosomiasis
ko03060: Protein export	0.021	0.006	
ko00750: Vitamin B6 metabolism	0.020	0.018	
**ko00020: Citrate cycle (TCA cycle)**	0.020	0.014	
**ko00195: Photosynthesis**	0.019	0.255	
ko00061: Fatty acid biosynthesis	0.017	0.008	
ko00770: Pantothenate and CoA biosynthesis	0.017	0.007	
**ko00450: Selenocompound metabolism**	0.015	0.026	
ko04122: Sulfur relay system	0.015	0.004	
**ko00720: Carbon fixation pathways in prokaryotes**	0.015	0.011	
ko00010: Glycolysis / Gluconeogenesis	0.015	0.023	
ko00970: Aminoacyl-tRNA biosynthesis	0.015	0.049	
**ko00030: Pentose phosphate pathway**	0.014	0.011	
ko03020: RNA polymerase	0.014	0.002	
**ko00730: Thiamine metabolism**	0.014	0.004	
ko00630: Glyoxylate and dicarboxylate metabolism	0.013	0.018	
**ko00620: Pyruvate metabolism**	0.013	0.018	
**ko00250: Alanine, aspartate and glutamate**	0.013	0.008	
**ko00260: Glycine, serine and threonine metabolism**	0.012	0.007	
**ko00785: Lipoic acid metabolism**	0.012	0.002	

The relative abundance of the pathway is shown for each pathway and host. Pathways also in the top 25 pathways predicted by PICRUSt^126^ and Tax4Fun^127^ are bolded.

### Functional equivalence in sponge and coral microbiomes

Reads and transcripts were identified for multiple pathways involved in elemental cycles, including carbon (C), nitrogen (N), and sulfur (S), in both host species. Previous studies have also highlighted the importance of major C and N pathways in sponge- and coral-microbe symbioses^18,53,70,71^. In the current study, there was often overlap in the presence of major pathways for nutrient acquisition and/or catabolism, with only a few specific pathways (i.e., DNRA, sulfur oxidation) exclusive to sponges or corals. We highlight here the complexity in comparing nutrient pathways at both a broad (e.g., “N cycling”) and fine-scale level (e.g., “ammonia oxidation pathway”), which makes generalizations more difficult, but the latter provides more biologically meaningful information. In terms of C fixation and catabolism, photosynthesis and oxidative phosphorylation were evident in both host species but were approximately 24% and 18% more abundant in corals, respectively (Fig. 5). Because of the differences detected in C metabolism in both the short-read analysis and assembly analysis we discuss this topic further in the “Specialization” section below.

**Figure 6:**
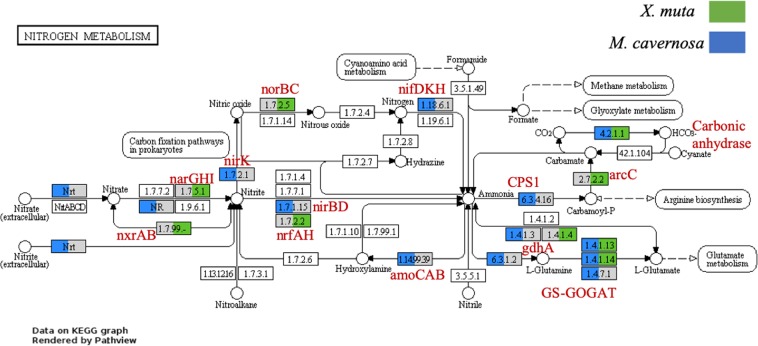
Nitrogen metabolism in the coral *Montastraea cavernosa* and sponge, *Xestospongia muta* microbiomes. Transcripts that mapped to nitrogen metabolism KEGG pathway^67^ are shown here with color representing the presence of one or more transcripts. Visualization performed with Pathview^122^. Gray color indicates no transcripts were mapped for one of the hosts, no color indicates no transcripts mapped for either host.

In contrast to central C metabolism, no pathways were identified as significantly different between host taxa for N metabolism based on the short-read analysis, potentially indicating high convergence in N cycling between host species similar to previous studies on sponges^53^. However, we note that the assembly analysis revealed some differences in presence of transcripts (Fig. 6). In both host species complete nitrification, which converts ammonia to nitrate, was not detected. Nitrification has been documented in corals previously^72,73^ and transcripts for the first step of ammonia oxidation were observed only for the *M. cavernosa* (Fig. 6). The lack of nitrification transcripts for *X. muta* was surprising as nitrification is known to occur in this sponge^57,59^. It may be that nitrification was not occurring at the time of sampling. In support of this, *Nitrospirae* bacteria known to contribute to nitrification were documented in the total community but were a minor portion of the active community (Supplementary Information S2). This result is supported by published studies documenting high variability in pumping activity in *X. muta*^58^ and suggests corresponding variability in nitrification activity, an area for further work.

Denitrification was also not detected in either host species. Dentrifying bacteria have been observed in coral^72,74^ and recently, denitrification rates were documented for three coral species in the Red Sea^71^. Thus, either denitrification does not occur in this coral, *M*. *cavernosa*, or it was not occurring at the time of sampling. In contrast, the sponge microbiome contained several, but not all steps of the denitrification pathway and genes involved in dissimilatory nitrate reduction to ammonia (DNRA), indicating the existence of anaerobic dissimilatory nitrogen processes in this host. There may be more opportunity for anaerobic microhabitats within the sponge host, due to variable pumping activity, than within the coral. However, sampling for this study was conducted in the mid-morning for *X. muta* and midday for *M. cavernosa* and it is possible that this is a factor in the differences detected here between host organisms. Furthermore, it is likely that some anaerobic metabolism could occur in the coral microbiome at night due to diurnal fluctuations in tissue *p*O_2_. However, spatial separation can also allow for simultaneous aerobic and anaerobic processes and *p*O2 was not found to influence denitrification and nitrogen fixation rates in Red Sea stony coral^71^. Dissimilatory metabolism thus appears to represent a mix of potential convergence (e.g., lack of significant differences based on short-read analysis) and some specialization (i.e., DNRA in *X. muta*) between host species.

Assimilation of N using glutamate dehydrogenase (*gdh*A), which is generally used during times of nitrogen excess^75^, was evident in both host species indicating a common form of N assimilation (Fig. 6). However, in *M. cavernosa*, transcripts were also recovered for the canonical glutamine synthetase and glutamate synthase (GS-GOGAT) pathway, indicating that at least at the time of sampling these microbes were not nitrogen limited. Interestingly, transcripts representing *nif*H, a component of the nitrogenase enzyme for nitrogen fixation were recovered only from *M. cavernosa*, highlighting the potential for ammonia concentration gradients within the coral host. These results support a critical role of nitrogen recycling with the holobiont.

**Figure 7:**
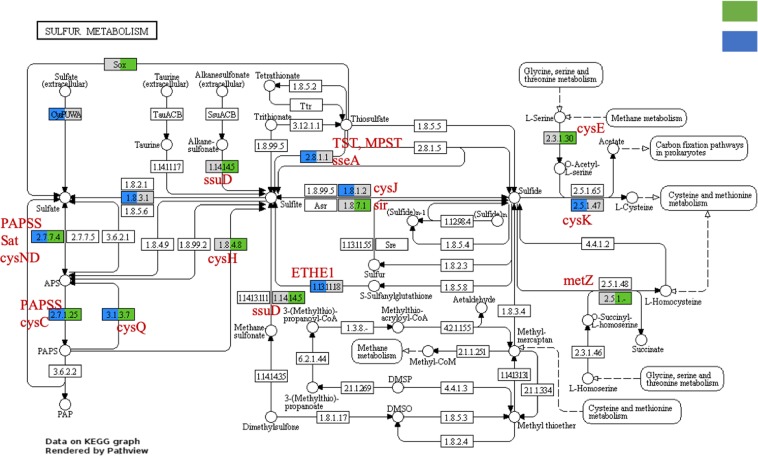
Sulfur metabolism in the coral*, Monstrastrea cavernosa* and sponge, *Xestospongia muta* microbiomes. Transcripts that mapped to sulfur metabolism KEGG pathway^67^ are shown here with color representing the presence of one or more transcripts. Visualization performed with Pathview^122^. Gray color indicates no transcripts were mapped for one of the hosts, no color indicates no transcripts mapped for either host.

In terms of S cycling, there was overlap in coverage of S related pathways in the two host species, although some pathways were more complete in *X. muta* than *M. cavernosa*. No transcripts corresponding to dimethylsulfoniopropionate (DMSP) production were observed in *M. cavernosa*, which was expected as this pathway is thought to be driven by Symbiodiniaceae^41^ and those eukaryotic transcripts were not included in this analysis. While both hosts expressed sulfate assimilation genes, only *M. cavernosa* expressed sulfate transport and only *X. muta* expressed sulfur oxidation genes and other sulfur metabolism genes such as alkanesulfonate monoxygenase (*ssu*D, Fig. 7). For example, sulfur assimilation by sulfate reduction to adenylyl sulfate (APS), 3′-phosphoadenylyl sulfate (PAPS), and to sulfide, was observed in each host microbiome. However, other steps in organic sulfur compound metabolism differed between the hosts, potentially indicating that these microbial communities utilize or have available to them different compounds depending on their host. Furthermore, the assimilation of sulfonates in *X. muta* microbiome could indicate sulfur-limiting conditions^76^.

Evidence of sulfur oxidation in microbiomes of other sponges^77,78^ corals^79^, and other invertebrates^80^ suggests this is a common energy source for symbiotic microbes, although it could be regulated by diurnal fluctuations in oxygen concentration in *M. cavernosa*. Transcripts for a thiosulfate transporter (*cys*P) were recovered from *M. cavernosa* (Fig. 7), which could represent an important source and means of obtaining sulfur for coral symbionts. Symbiotic bacteria in other systems also show elevated expression of genes related to sulfur uptake and metabolism in the symbiotic state relative to the free-living state^76^; this may represent an important trait in symbiotic bacteria.

Pathway predictions (Tax4Fun and PICRUSt) highlighted several other major pathways with overlap between host species although there were some differences in pathway abundance. This high overlap occurs despite deep phylogenetic differences in the host taxa and differences in sampling location and time. The similar pathways include vitamin metabolism ketone body metabolism, phosphonate and phosphinate metabolism, and siderophore production. Vitamin metabolism has been highlighted as an important trait for sponge-microbe symbioses^53,70^ and may be critical for stability of symbiosis under environmental stress such as ocean acidification^70^. Our results indicate that despite differences in host taxa, vitamin metabolism remains an abundant and likely important metabolic pathway for symbiont microbiomes. Transcripts for ketone body metabolism were present in both microbiomes but were more abundant in the coral microbiome (Table 3, Supplementary Fig. S6). Diverse bacteria can produce and degrade ketone molecules either anaerobically^81^ or aerobically^82^. In addition to their role in central metabolism, ketones have been of interest for years as bioactive compounds from soft corals and sponges^83,84^. *Montastraea cavernosa* does contain both aqueous and organic compounds that deter feeding by fish^85^, which could be produced by the microbiome, although these compounds have not been specifically characterized.

*Montastraea cavernosa* and *X. muta* both contained prokaryotic pathways of phosphonate and phosphinate metabolism (Fig. 4). Pathway coverage was higher in *M. cavernosa*, which is intriguing as corals and dinoflagellates (i.e., Symbiodiniaceae symbionts of corals) use phosphonates, which contain a phosphorus-carbon bond, in lipid metabolism to form phosphonolipids in cell membranes^86–88^. Phosphonate compounds are an abundant component of the dissolved organic phosphorus pool in marine systems^89–91^, but inorganic phosphorus is typically very low in concentration in coral reef seawater^92,93^. Thus, host-derived phosphonate compounds may be an important source of phosphorus for the coral microbiome community. Indeed, at least 13 coral species produce phosphonate and their associated microbes possess genes for phosphonate degradation^42^. Here, we add to these data by demonstrating not just presence but expression of phosphonate metabolizing genes in the coral holobiont. Furthermore, to our knowledge, there is limited information available on the presence and activity of phosphonate compounds and metabolism in invertebrates^87,93^ (and references therein). However, phosphonate metabolism is prevalent in marine microbes^91^ and our results here as well as another recent report of phosphonate metabolism in sponges^94^, indicate at least some metabolic activity of phosphonate compounds by sponge microbes.

Lastly, siderophore production was present in both host species but exhibited higher coverage in the coral than in the sponge microbiomes (Fig. 4). Iron is generally very low in concentration in the ocean and present in an insoluble form not bioavailable to aerobic organisms^95,96^. Many microbes produce siderophore compounds that chelate iron and are then taken up by the microbe. Siderophore production is a common feature of symbionts, including those of plants^97^, where it can facilitate iron acquisition and growth of the host^98,99^. Conversely, this trait is also common in pathogens, in which siderophores are used to sequester iron from the host or other organism^100^. An increase in genes related to siderophore production has been observed in bacteria considered to be potential coral pathogens^101^. While sponge associated microbes are also known to produce siderophores^70,102^, it may be that the increased volume of seawater pumped through sponges and the density of microbes within the sponge provide more opportunities to “cheat” and obtain iron-containing siderophores produced by other microbes. Coral mucus layers may also limit the diffusion of nutrients from seawater to symbionts.

### Specialization within the sponge and coral microbiomes

The short-read analysis of *X. muta* and *M. cavernosa* metatranscriptomes and the metabolic pathway predictions based on 16S rRNA gene profiling of the coral and sponge^55,60^ predicted higher abundance of xenobiotic metabolism (e.g., atrazine, nitrotoluene degradation) in sponges than in corals (Table 3, see Supplementary Information S1 for further discussion of metagenome prediction). The term ‘xenobiotic’ is used in KEGG ontology and refers to compounds not made by the organism itself, although in our dataset it is possible that some of these compounds are produced by the host or associated microbial community. In the short-read analysis, this difference between host species was supported by both pathway coverage and abundance (i.e., chloroalkane degradation, Fig. 4, Supplementary Fig. S4). Furthermore, the unique metabolic pathways observed in *X. muta* based on short-read analysis included several xenobiotic metabolic pathways (Table 3). Thus, multiple lines of evidence highlight the biosynthetic and catabolic potential of a range of secondary metabolites as important pathways in the sponge holobiont. Sponges and corals are well known for the diverse metabolism of their microbial symbionts and degradation of xenobiotic compounds^103–105^, including terpenoid compounds observed to be more abundant in pathway abundance in *X. muta* (Fig. S4). *Xestospongia muta* in particular, is known to produce diverse secondary metabolites including halogenated compounds^106,107^. Sponges are also notable for their ability to degrade diverse compounds and have even been used as bioremediation agents for organic pollutant^103,108^. Moreover, we also observed higher abundance of transcripts involved in fatty acid metabolism, a major pathway with established links to secondary metabolite metabolism^109,110^. The extent of differences in secondary metabolite or xenobiotic metabolism between *M. cavernosa* and *X. muta* is still difficult to quantify as there is a strong need for better characterization of such compounds and metabolic pathways. Additionally, *X. muta* was sampled from three different locations within the Caribbean and *M. cavernosa* from one of those locations, and we do not have data on the presence of these compounds at each location. Our initial hypothesis at least, is that xenobiotics or holobiont-derived metabolites are likely to be present at low concentrations in seawater and as sponges filter large volumes of seawater through their bodies^58,91^, the resident microbes, and sponge cells, may have a greater need to metabolize these compounds compared to microbes in corals.

We suggest that photosynthesis, to some extent, is a specialized trait within the coral microbiome. While the presence of photosynthesis and C-fixation pathways were expected as both host species harbor photoautotrophic symbionts. The elevated abundance of reads that mapped to the photosynthesis pathway (Fig. 5) in coral prokaryotic microbiomes was surprising, as this process is generally attributed to the Symbiodiniaceae in corals. Further, the density of microbes is likely to be lower in *M. cavernosa* than in *X. muta*, a high microbial abundance sponge^111^, underscoring the significance of our results. *Cyanobacteria* and other phototrophic bacteria (e.g., certain clades of *Proteobacteria* and *Chloroflexi*) within coral tissues^28,112^ or trapped in mucus^113–115^, as well as recently described symbiotic apicomplexan protists^116^ may have contributed to elevated abundance of genes associated with photosynthetic pathways in the *M. cavernosa* microbiome samples and could be an important energy source for the microbiome in healthy corals. Some sponges harbor Symbiodiniaceae as symbionts, however, *X. muta* is not known to contain this dinoflagellate as a symbiont and the transcripts identified here likely come from *Cyanobacteria*, which constitute a major proportion of the microbial community in *X. muta*^60,117,118^. Similarly, transcripts for carbon fixation in photosynthetic organisms were also more abundant in the coral than sponge microbiome (Supplementary Fig. S5), supporting the significantly higher abundance of this pathway in coral in the short-read analysis.

Another central energy pathway, oxidative phosphorylation, was also present in both host species but elevated in *M. cavernosa* (Fig. 5). Genes for respiration in *M. cavernosa* were also noted as highly abundant in previous metagenome work comparing microbiomes of nine different biomes^54^, similar to our results presented here. This could reflect high heterotrophic metabolism in microbes utilizing coral mucus, a phenomenon described previously^115,116^. However, the difference abundance of oxidative phosphorylation pathways between *M. cavernosa* and *X. muta* is somewhat surprising as heterotrophic metabolism is also expected in *X. muta* based on the microbial community composition^115,119^ and this pathway was highlighted previously as abundant in sponge microbial communities^70^. Further genomic work is needed to elucidate if, and what are the biological relevance of such differences between the *M. cavernosa* and *X. muta* communities.

## Conclusions

Our data support the evolutionary convergence in the microbiome functions of the coral *Montastraea cavernosa* and the sponge, *Xestospongia muta*, highlighted here by the few significant differences in functional gene profile based on short-read analysis. Despite the presence of Symbiodiniaceae in *M. cavernosa* and absence in *X. muta*, there is significant overlap in many KEGG prokaryotic cellular and metabolic pathways. In particular, similarities in N (e.g., nitrification and nitrogen assimilation via glutamate dehydrogenase), P (e.g. phosphonate metabolism), and S (e.g., sulfate reduction) cycling were observed based on short-read analysis of mRNA. A similar observation has also been made across sponge taxa^62,120–122^ and in other microbiome systems^64,123–125^, supporting the notion that conservation of function rather than taxonomy is a common characteristic of symbiont communities. To our knowledge, however, functional convergence has not been specifically investigated within coral hosts. Further, we also observed differences in symbiont diversity and pathway coverage that may underlie specialized functional characteristics of each microbiome reflective of distinct host-microbe interactions. Surprisingly, photosynthesis and C-fixation were more abundant in *M. cavernosa* which also hosts Symbiodiniaceae while secondary metabolite metabolism, and potentially related pathways (e.g., fatty acid metabolism) appeared to be of more significance in *X. muta*. We propose several additional questions for further research that our results have highlighted. One is that there are no metatranscriptome studies that have examined diurnal differences in the coral microbiome, but such work could shed light on oxygen-dependent microbial activity. Similarly, while dentification was not detected in *M. cavernosa*, it would be worth investigating this process further as this coral species hosts N-fixing bacteria and the two N processes were recently shown to be linked in coral^70^. Separately, phosphorus cycling is of recent interest in holobiont metabolism^122,126,127^, and specifically, the role of Symbiodiniaceae in the production of phosphonates and whether this source of phosphorus is available to microbes associated with sponges (with or without Symbiodiniaceae), may be fruitful questions for future work. This study sheds new light on the functional diversity of sponge and coral microbiomes and reveals potential metabolic specialization of microbiomes within different invertebrate hosts.

## Methods

### Sample collection, sequencing, and assembly

The sponge samples of *Xestospongia muta* (n = 3) were collected one each from three Caribbean locations: Conch Reef in the Florida Keys, Rock Bottom Reef near Little Cayman, Cayman Islands, and North Perry Reef near Lee Stocking Island, Bahamas in July and August 2011 as described in^17^. The coral samples of brown and orange morph of *Montastraea cavernosa* (n = 3 each) were collected from North Perry Reef near Lee Stocking Island in the Bahamas in August of 2011 and processed as previously described^55^. All samples were collected from approximately 15 m depth; however, *X. muta* samples were collected in the morning (09:00) while the *M. cavernosa* samples were collected around midday. DNA and RNA analyses were conducted with these samples. At each location, maximum photosynthetically active radiation (PAR; 400700 nm) irradiance is similar, ~500–600 µmol quanta m^−2^ s^−1^ at noon at each location approx. 15 m depth (M.P. Lesser, unpublished data). There were, however, statistically significant differences in concentrations of NO_3_^−^ (ANOVA, F_3,23_ = 3.5, *p* = 0.02, range 0.05–1.2 µM), with the highest average at Little Cayman (0.8 µM ± 0.3). There were no difference in concentrations of NH_4_^+^ across locations (ANOVA, F_3,3.8_ = 1.1, *p* = 0.3, data from Fiore *et al*.^59^).

For marker gene profiling, DNA was extracted, 16S rRNA genes were amplified with PCR, and amplicons were sequenced with 454 pyrosequencing and analyzed using the same protocol within the QIIME (v1) pipeline^128^ as described previously^55,60^. Methods for OTU generation did not differ significantly between the sponge and coral; identical primers, PCR setup, and initial processing in QIIME to generate OTUs clustered at 97% identity following removal of singleton reads. However, for analysis in this study the OTU sequences from the previous studies^55,60^ were reclassified using the SILVA^129^ release 132.

For metatranscriptome analysis for both coral and sponge, total RNA was extracted, eukaryotic rRNA was removed by subtractive hybridization with a RiboMinus Eukaryote kit (Invitrogen), and reverse transcribed RNA was sequenced using an Illumina HiSeq. 2000 as described previously^17,55^. The raw RNA reads from each sample were quality trimmed and then used in two separate analyses; short-read analysis of putative mRNA reads and metatranscriptome assembly, forming contiguous sequences (contigs) from the putative mRNA reads. First, in the short-read analysis, the reads were then mapped to SILVA database v.111 and the human genome to remove rRNA and human contamination respectively, using the mapx algorithm by Real Time Genomics (RTG, www.realtimegenomics.com). The remaining unmapped reads, enriched for mRNA, were mapped using the mapx algorithm to approximately ~5000 prokaryotic genomes in the IMG database (available as of February 2013) (https://img.jgi.doe.gov/). The reads that mapped to IMG were considered putative prokaryotic mRNA reads and were analyzed for functional annotation with the HUMAnN pipeline^66^ (v.1) using Kyoto Encyclopedia of Genes and Genomes (KEGG^67^). HUMAnN was designed to use metagenomic data to produce a normalized estimate of coverage (presence/absence) and abundance for KEGG metabolic pathways. The pathway coverage, where a pathway consists of two or more genes in that pathway^66^ and abundance (relative abundance per sample) were calculated for all samples. The combined pathway coverage and abundance files were analyzed with STAMP^68^ as described in the main text. Multiple comparisons between the three sample types were calculated using analysis of variance (ANOVA) with Benjamin-Hochberg FDR correction for *p*-values^69^, and an effect size of 0.8. Pairwise comparisons between corals and sponges were performed using Welch’s *t*-test with Benjamin-Hochberg FDR correction for *p*-values and a relative effect size (i.e., the magnitude of difference between groups^68^) in the ratio proportions of the two groups of 2. In those pairwise comparisons of pathway abundance between sponge and corals, a minimum effect size of 0.8 in the difference between proportions was required, to reduce the number of low abundance pathways that were significantly different but unlikely to be biologically meaningful. Heatmaps were created in STAMP based on significant differences from the multiple comparison analysis. Scatter plots and extended error plots were created in STAMP using mean proportions of abundance or coverage data.

Second, we performed metatranscriptome assembly using quality trimmed RNA reads that did not map to SILVA or to host genomes^17,55^ to produce putative mRNA transcripts using Trinity^130^ as previously described^17,55^. The same assemblies were used for analysis in the current study, but only the Trinity assembly was used for the sponge metatranscriptome^17^ to be consistent with assembly methods for the corals^55^. Sponge RNA reads were quality filtered based on average quality score of 30, a minimum length of 50 nt, removal of adapter sequence and an initial “N” at the start of each reads, and unpaired reads were removed. Briefly, the sponge metatranscriptome was assembled with Trinity and sponge host contiguous sequences (contigs) were distinguished from microbial contigs based on MEGAN^131^ analysis as previously described^19^. Coral metatranscriptome contigs were assembled with Trinity and BinPacker (https://goo.gl/Q8pWUu), then separated into coral host, *Symbiodinium*, and microbial community bins by BLASTX comparison to a custom database comprising RefSeq protein data sets from plants, Bacteria, Archaea, Fungi, Protozoa, and Invertebrates, and a proteome from *Symbiodinium*^21^. The main difference between the sponge and coral assemblies is that a more stringent quality score threshold (Phred <2) was used for corals than for sponges (<30). Here we compare these microbial metatranscriptomes in a transcript presence/absence context only as a means to supplement the short-read analysis. Read and assembly data are available as previously described^17,55,60^ (iMicrobe CAM_P_0000957 (454 pyrosequencing data) and CAM_P_0001214 (metatranscriptome data) sponge; European Nucleotide Archive ID PRJEB18062 (454 pyrosequencing) and DRYAD 10.5061/dryad.v2g01 (metatranscriptome) coral).

### Comparison of total and active microbial communities

The ‘total’ microbial community was characterized using previously published data of pyrosequencing of 16S rRNA amplicons as described above and in previous work^55,60^, except that sponge samples were subsampled to a depth of 2200 reads per sample for this study to enable direct comparison with coral samples. The taxonomy of the metabolically ‘active’ microbial community (i.e., those producing RNA) was characterized by mapping putative mRNA reads to reference prokaryotic genomes in the IMG database, as described above. The taxonomic identity the top hit was captured by the mapx algorithm (see Supplementary Information S2).

### Metagenome prediction based on 16S rRNA gene profiling and comparison to metatranscriptome assembly

To complement the metabolic ‘snapshot’ of the two holobionts generated by the metatranscriptomes and to test the robustness of 16S rRNA-based community function predictions, we compared the metatranscriptome-derived KEGG orthology (KO) to those predicted by 16S rRNA gene profiling. For metagenome prediction, we used the quality trimmed 16S rRNA reads described above with two published programs: PICRUSt v.1.1.3^132^ and Tax4Fun v.0.3.1^133^. PICRUSt predictions were performed using the precalculated files for the Greengenes v13.5 OTU taxonomy. First, the 16S rRNA OTU table was normalized using the *normalize_by_copy_number.py* script. Secondly, metagenome functional predictions and weighted nearest sequenced taxon index (NSTI) scores for each sample were created using the *predict_metagenomes.py* script. Tax4Fun predictions were performed using the SILVA database (v123) database for QIIME provided by the developer team (http://tax4fun.gobics.de/). The fraction of taxonomic units unexplained (FTU) scores by measuring the fraction of sequences assigned to taxonomic units that cannot be mapped to KEGG organisms using the Tax4Fun association matrix. The NSTI and FTU scores serve as a proxy for quality of the respective functional prediction, with a lower score indicating high similarity or correlation between the query 16S rRNA gene sequences and the reference genomes^132,134^. Metagenomic functional profiles were calculated using the standard parameters (Tax4Fun parameters: refProfile = “UProC”, shortReadMode = FALSE, normCopyNo = TRUE).

The resulting KO abundance tables from each prediction were processed with HUMAnN (v.1) to produce pathway coverage estimates for each sponge and coral sample based on KO as described above for the metatranscriptome short-read analysis. Because differences in functional gene expression may not be captured by 16S rRNA gene data, we consider HUMAnN-derived pathway coverage to be a more comparable value than pathway abundance between the metagenome prediction and metatranscriptome data. However, we present both pathway coverage and abundance comparisons, and focus the comparative analysis using pathway coverage.

The predicted KOs were compared with those generated by the metatranscriptome read mapping as described below (under *Functional exploration of metatranscriptomes*). Pearson correlations between the pathway coverage of KOs from the predictions and pathway coverage from the metatranscriptome were performed in R v3.4.0 (R Core Team, 2017), while further community-level comparisons were performed with STAMP v2.1.3^68^.

### Functional exploration of metatranscriptomes

Functions and metabolic pathways of interest were identified in metatranscriptomes using both the short-read analysis to provide an overview and assembly method to provide more in-depth analysis of specific metabolic pathways. For the functional overview, we used STAMP to identify all pathways that were differentially present, or differentially expressed, between corals and sponges or between the color morphs of the coral. This analysis was based on the short-read analysis processed with the HUMAnN pipeline (v.1) as described above. The HUMAnN-derived pathway coverage and abundance from each sample were concatenated to form separate ‘OTU-type’ tables of coverage and abundance for analysis with STAMP. In STAMP, principal components analysis (PCA) was performed and multiple comparisons between the three sample types were calculated using analysis of variance (ANOVA) with an effect size threshold of 0.8 and Benjamin-Hochberg FDR correction for *p*-values (see Supplementary Information S1).

For the assembly-based investigation of certain metabolic pathways, we selected key elemental cycles and processes on coral reefs based on a review of the literature, including photosynthesis and carbon fixation, the nitrogen cycle, the sulfur cycle, and degradation of xenobiotics, and searched for these metabolic pathways in the metatranscriptome annotations as described below. In this analysis, we used the assembled contiguous sequences (contigs) derived from Trinity algorithms^17,55^ and performed a reanalysis in order to uniformly compare the coral and sponge microbial communities. The RNA pools of *M. cavernosa* and *X. muta* were extracted at the same time, sequenced on the same Illumina HiSeq lane, and were assembled using Trinity, although settings were optimized for each dataset. Thus, while there are caveats such as differences in assembly settings and differences in sampling time between the two assemblies, the comparisons here which are limited to presence/absence of transcripts in metabolic pathways of interest, should be robust enough for foundational comparisons. Assembled transcripts from coral and sponge were translated into protein sequences with prodigal (v2.6.3) using the -meta flag, and KO numbers were assigned to proteins (genus_prokaryotes + family_eukaryotes) with GhostKOALA^133^. Proteins present from each pathway of interest were collected and the resulting pathway files were viewed in Pathview^135^ to compare presence/absence of KOs for each microbiome community. The use of assembled contigs here allowed us to capture visualize the presence and taxonomic identity of transcripts that correspond to specific genes within metabolic pathways of interest. Such fine-scale information was not possible with the short-read analysis processed with the HUMAnN pipeline.

## Supplementary information

Supplementary Information S1.

Supplementary dataset S2.
