# Supplementary material for: Trait-Based Comparison of Coral and Sponge Microbiomes
Source: Sci Rep. 2020 Feb 11;10:2340. doi: 10.1038/s41598-020-59320-9 (PMC7012828; doi:10.1038/s41598-020-59320-9)
Supplement: Supplementary file 1 — Supplementary Information S1. [file 41598_2020_59320_MOESM1_ESM.docx]

**Trait-Based Comparison of Coral and Sponge Microbiomes:**

*Supplemental information*

Cara L. Fiore^1,2,3^, Jessica K. Jarett^1,2,4^, Georg Steinert^5,6^, Michael P. Lesser^1^

^1^University of New Hampshire, Department of Molecular, Cellular and Biomedical Sciences, School of Marine Science and Ocean Engineering, Durham NH, USA

^2^Authors contributed equally to this work

^3^Current address: Appalachian State University, Biology Department, Boone NC, USA

^4^Current address: AnimalBiome, Oakland CA, USA

^5^Institute for Chemistry and Biology of the Marine Environment, Carl-von-Ossietzky University Oldenburg, Wilhelmshaven, Germany

^6^Current address: GEOMAR Helmholtz Centre for Ocean Research Kiel, Marine Symbioses, Kiel, Germany

Below are expanded results & discussion and methods, as well as six supplemental figures and four tables. Note that supplementary information S2 contains the OTU table as an excel file.

**Supplementary Results and Discussion**

*Comparison of total and active microbial communities*

As lower-level taxonomic assignments are difficult to derive confidently from read-level mapping, we compared the communities between the coral and sponge hosts only at the phylum level (Supplementary file S2). In general, there was a greater difference between the active vs. the total community in the corals as compared to the sponges (Fig. 2; Supplementary Fig. S1; Supplementary information S2). This may in part be due to diurnal differences in oxygen concentration in the coral, driven primarily by photosynthesis, that could allow some microbes to be more metabolically active during the day whereas others are active at night.^1^ The environment within sponges can also contain gradients in oxygen concentration,^2,3^ but these are correlated with variation in sponge pumping activity rather than being diurnal. In total, the two coral morphs were largely similar and resembled each other much more closely than they resembled the sponge microbial community. It is difficult to compare abundance between the hosts as these data are relative abundances, however, we observed several interesting patterns that may be useful for future work. The *Alphaproteobacteria* group was a higher proportion of the total community than the active community in the corals but vice versa in sponges (Supplementary information S2), supporting previous work that highlights the metabolic and ecological relevance of this diverse group of bacteria in sponges^4–8^. In contrast, the *Bacteroidetes* comprised a higher proportion of the total and active communities in the corals relative to the sponges (Supplementary Fig. S1; Supplementary information S2). *Bacteroidetes* have been observed in corals^9,10^ and sponges,^11,12^ and are often considered transient or potentially pathogenic taxa in both hosts,^13–15^ yet this group may be an important member of the coral microbiome and may represent a group that would be of interest for further genomic investigations. We further suggest that quantitative microbiome profiling^16^ will be crucial for future comparative microbiome analysis.

**Supplemental Methods**

*Metagenome prediction based on 16S rRNA gene profiling*

In addition to comparing the total to the active microbial community based on taxonomy, we predicted community function from 16S rRNA with both PICRUSt^17^ and Tax4Fun^18^ and compared this to the sequenced metatranscriptome (Table 2). Some pathways were predicted but not observed in the metatranscriptome, such as aromatic compound degradation, steroid biosynthesis, some secondary metabolite biosynthesis, and canonical pathways such as mRNA surveillance pathway and NOD-like receptor signaling pathway (Supplementary Tables S3 (PICRUSt), S4 (Tax4Fun)). These could represent pathways that were not highly expressed enough to be captured at this sequencing depth or could be over-predicted by the programs (i.e., incorrectly predicted to be expressed). Few pathways were observed in the metatranscriptome and not predicted by either program (Supplementary Tables S3, S4).

The predicted metabolic pathways with the highest coverage were largely overlapping between the sponge and coral microbiomes (Table S2), including amino acid metabolism, central carbon metabolism, some fatty acid and secondary metabolite metabolism, and bacterial chemotaxis (Table S2). In addition to their role in the central metabolism of prokaryotes, these pathways have been characterized in sponge and coral microbiomes previously and may represent key functional pathways in microbiomes^19-21^. Functional similarity in certain traits, particularly related to nutrient cycling, across taxonomically distinct microbial communities has been documented in many sponge hosts^19,22^ and is now a well-established paradigm in the gut microbiome literature^23-24^. For example, a significant correlation was detected between metagenome function and diet in bats but not between diet and the taxonomic composition of the microbiome, suggesting that there is a selective force for metabolic function rather than taxonomic identity of microbiome members^24^. Taken together, our data support the notion that there are functional traits common to host-associated microbes, even across host taxa of different phyla.

**References**

1. Kühl, M., Cohen, Y., Dalsgaard, T., Jergensenl, B. B. & Revsbech, N. P. Microenvironment and photosynthesis of zooxanthellae in scleractinian corals studied with microsensors for O_2_, pH and light. *Marine Ecology Progress Series* **117,** (1995).

2. Hoffmann, F. *et al.* An Anaerobic World in Sponges. *Geomicrobiol. J.* **22,** 1–10 (2005).

3. Lavy, A., Keren, R., Yahel, G. & Ilan, M. Intermittent Hypoxia and Prolonged Suboxia Measured In situ in a Marine Sponge. *Front. Mar. Sci.* **3,** 263 (2016).

4. Enticknap, J., Kelly, M., Peraud, O., & Hill, R.T. Characterization of a culturable alphaproteobacterial symbiont common to many marine sponges and evidence for vertical transmission via sponge larvae. *Am Soc Microbiol.* **72**,3724-3732 (2006).

5. Sharp, K., Eam, B., Faulkner, D.J. & Haygood, M.G. Vertical transmission of diverse microbes in the tropical sponge *Corticium* sp. *Am Soc Microbiol.* **73**, 622-629 (2007).

6. Kim, T. K. & Fuerst, J. A. Diversity of polyketide synthase genes from bacteria associated with the marine sponge Pseudoceratina clavata: culture-dependent and culture-independent approaches. *Environ. Microbiol.* **8,** 1460–1470 (2006).

7. Lafi, F. F., Garson, M. J. & Fuerst, J. A. Culturable Bacterial Symbionts Isolated from Two Distinct Sponge Species (Pseudoceratina clavata and Rhabdastrella globostellata) from the Great Barrier Reef Display Similar Phylogenetic Diversity. *Microb. Ecol.* **50,** 213–220 (2005).

8. Mohamed, N. M. *et al.* Diversity and quorum-sensing signal production of Proteobacteria associated with marine sponges. *Environ. Microbiol.* 1**0,** 75-86 (2008).

9. Sweet, M. J., Croquer, A. & Bythell, J. C. Bacterial assemblages differ between compartments within the coral holobiont. *Coral Reefs* **30,** 39–52 (2011).

10. Meron, D. *et al.* The impact of reduced pH on the microbial community of the coral Acropora eurystoma. *ISME J.* **5**, 51-60 (2011).

11. Hentschel, U., Piel, J., Degnan, S. M. & Taylor, M. W. Genomic insights into the marine sponge microbiome. *Nature Rev. Microbiol.* (2012).

12. Fiore, C. L., Jarett, J. K. & Lesser, M. P. Symbiotic prokaryotic communities from different populations of the giant barrel sponge, *Xestospongia muta*. *Microbiologyopen* **2,** 938–952 (2013).

13. Webster, N. S., Xavier, J. R., Freckelton, M., Motti, C. A. & Cobb, R. Shifts in microbial and chemical patterns within the marine sponge *Aplysina aerophoba* during a disease outbreak. *Environ. Microbiol.* **10,** 3366–3376 (2008).

14. Sekar, R., Kaczmarsky, L. & Richardson, L.L. Microbial community composition of black band disease on the coral host *Siderastrea siderea* from three regions of the wider Caribbean. *Mar. Ecol. Prog. Ser.* **362**, 85-98 (2008).

15. Barneah, O., Ben-Dov, E., Kramarsky-Winter, E. & Kushmaro, A. Characterization of black band disease in Red Sea stony corals. *Environ. Microbiol.* **9,** 1995–2006 (2007).

16. Vandeputte, D. *et al.* Quantitative microbiome profiling links gut community variation to microbial load. *Nature* **551,** 507 (2017).

17. Langille, M. G. I. *et al.* Predictive functional profiling of microbial communities using 16S rRNA marker gene sequences. *Nat. Biotechnol.* **31,** 814–821 (2013).

18. Aßhauer, K. P., Wemheuer, B., Daniel, R. & Meinicke, P. Tax4Fun: predicting functional profiles from metagenomic 16S rRNA data. *Bioinformatics* **31,** 2882–2884 (2015).

19. Fan, L. *et al.* Functional equivalence and evolutionary convergence in complex communities of microbial sponge symbionts. *Proc. Natl. Acad. Sci. U. S. A.* E1878–E1887 (2012).

20. Daniels, C. A. *et al.* Metatranscriptome analysis of the reef-building coral Orbicella faveolata indicates holobiont response to coral disease. *Front. Mar. Sci.* **2,** 62 (2015).

21. Tout, J. *et al.* Redefining the sponge-symbiont acquisition paradigm: sponge microbes exhibit chemotaxis towards host-derived compounds. *Environ. Microbiol. Rep.* **9,** 750–755 (2017).

22. Ribes, M. *et al*. Functional convergence of microbes associated with temperate marine sponges. *Environ. Microbiol.* **14**, 1224-1239 (2012).

23. Consortium, T. H. M. P. *et al.* Structure, function and diversity of the healthy human microbiome. *Nature* **486,** 207–214 (2012).

24. Phillips, C. D. *et al.* Microbiome Structural and Functional Interactions across Host Dietary Niche Space. *Integr. Comp. Biol.* **57,** 743–755 (2017).

25. Kanehisa, M. & Goto, S. KEGG: Kyoto encyclopedia of genes and genomes. *Nucleic Acids Res.* **28,** 27-30 (2000).

26. Abubucker, S. *et al.* Metabolic Reconstruction for Metagenomic Data and Its Application to the Human Microbiome. *PLoS Comput. Biol.* **8,** e1002358 (2012).

27. Parks, D. H., Tyson, G. W., Hugenholtz, P. & Beiko, R. G. STAMP: statistical analysis of taxonomic and functional profiles. *Bioinformatics* **30,** 3123–3124 (2014).

28. Luo, W., Pant, G., Bhavnasi, Y.K., Blanchard, S.G., Brouwer, C. Pathview web: user friendly pathway visualization and data integration. *Nucl acids Res* **45**, W501-508 (2017).


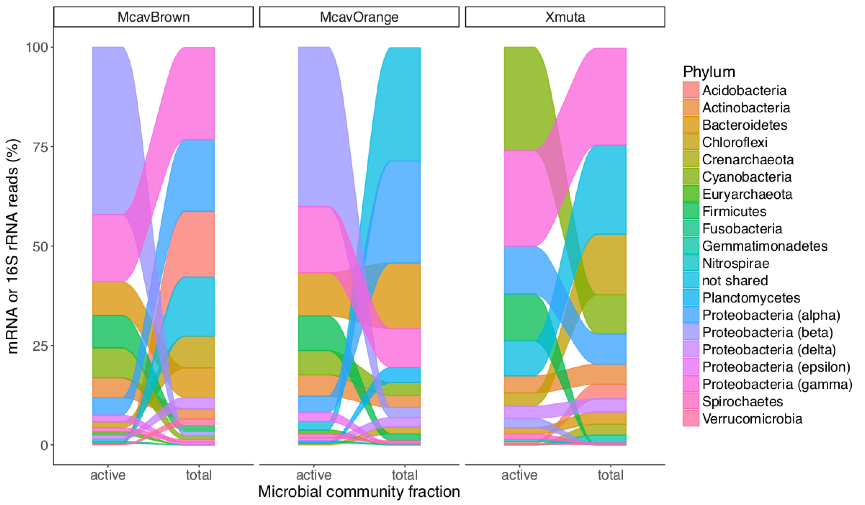


Figure S1. Comparison of taxa comprising active microbial communities, quantified by mapping of mRNA reads to a reference database, and total microbial communities, quantified with 16S rRNA PCR amplicon sequencing, in corals (*Montastraea cavernosa* (Mcav) brown (Br) and orange (Or) morphs) and sponges (*Xestospongia muta*). Phyla (or classes in the case of *Proteobacteria*) are ordered by abundance in each fraction and sample type.


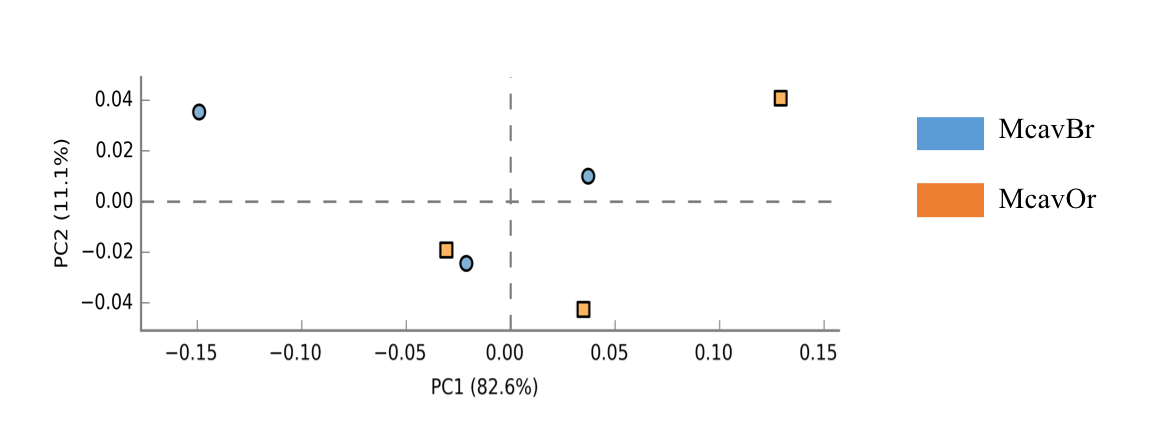


*M. cavernosa* Br

*M. cavernosa* Or

Figure S2. Principal components analysis (PCA) of KEGG Orthology pathways^25^ based on relative abundance using the coral, *Montastraea cavernosa* (brown (Br) and orange (Or) morphs). KEGG Orthology pathways were assigned, and relative abundance was calculated, with HUMAnN^26^ based on putative mRNA reads from the prokaryotic community. Visualized with STAMP^27^.


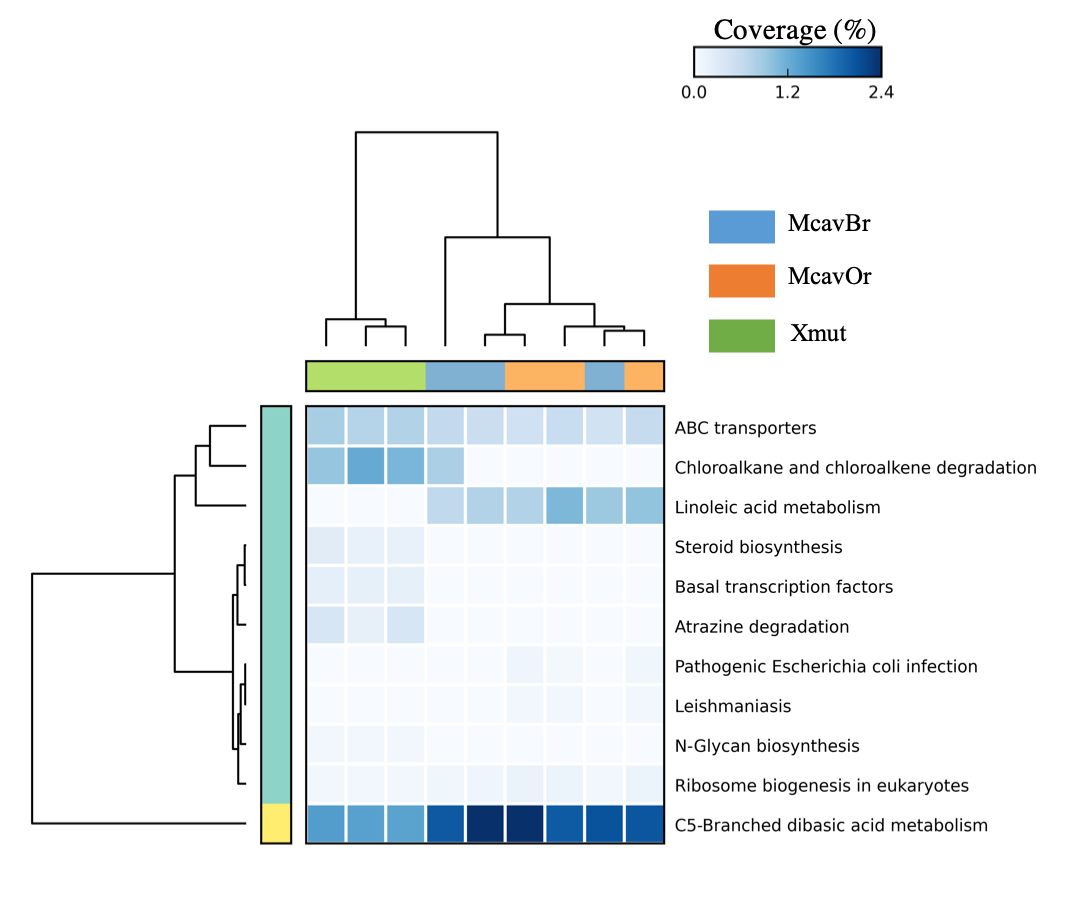


*M. cavernosa* Or

*M. cavernosa* Br

*X. muta*

Figure S3. Heatmap of pathway coverage in the brown (Br) and orange (Or) color morphs of the coral, *M.* *cavernosa* and the sponge, *X. muta*. Only pathways with significantly different coverage between sample types are shown resulting from multiple comparison testing by analysis of variance (ANOVA). KEGG Orthology pathways^25^ were assigned, and coverage was calculated, with HUMAnN^26^ based on putative mRNA reads from the prokaryotic community. Color bar on vertical dendrogram corresponds to clusters by hierarchical clustering. Visualized with STAMP^27^.


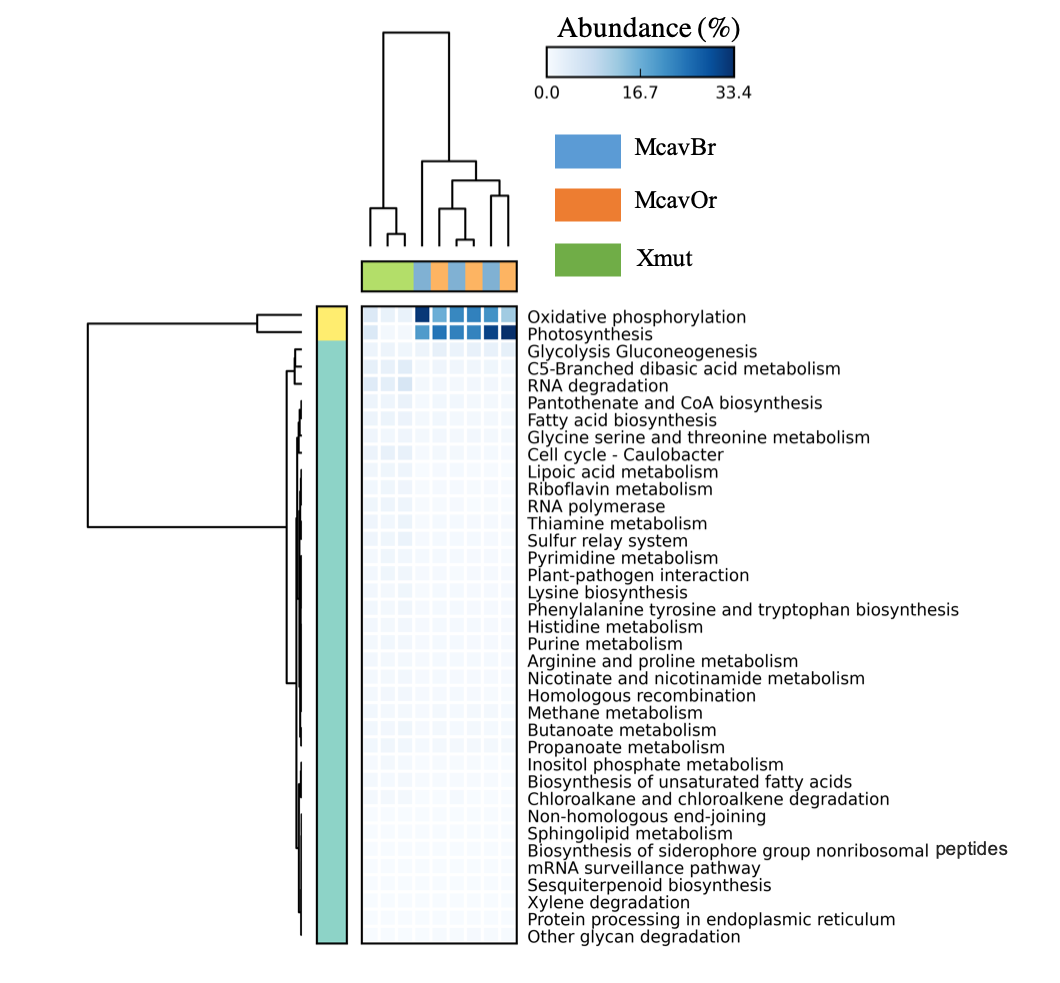


*M. cavernosa* Or

*M. cavernosa* Br

*X. muta*

*X. muta*

*M. cavernosa* Br

*M. cavernosa* Or

Figure S4. Heatmap of pathway relative abundance in the brown (Br) and orange (Or) color morphs of the coral, *M.* *cavernosa* and the sponge, *X. muta*. Only pathways with significantly different abundance between sample types are shown resulting from multiple comparison testing by analysis of variance (ANOVA). KEGG^25^ Orthology pathways were assigned, and relative abundance was calculated, with HUMAnN^26^ based on putative mRNA reads from the prokaryotic community. Color bar on vertical dendrogram corresponds to clusters by hierarchical clustering. Visualized with STAMP^27^.


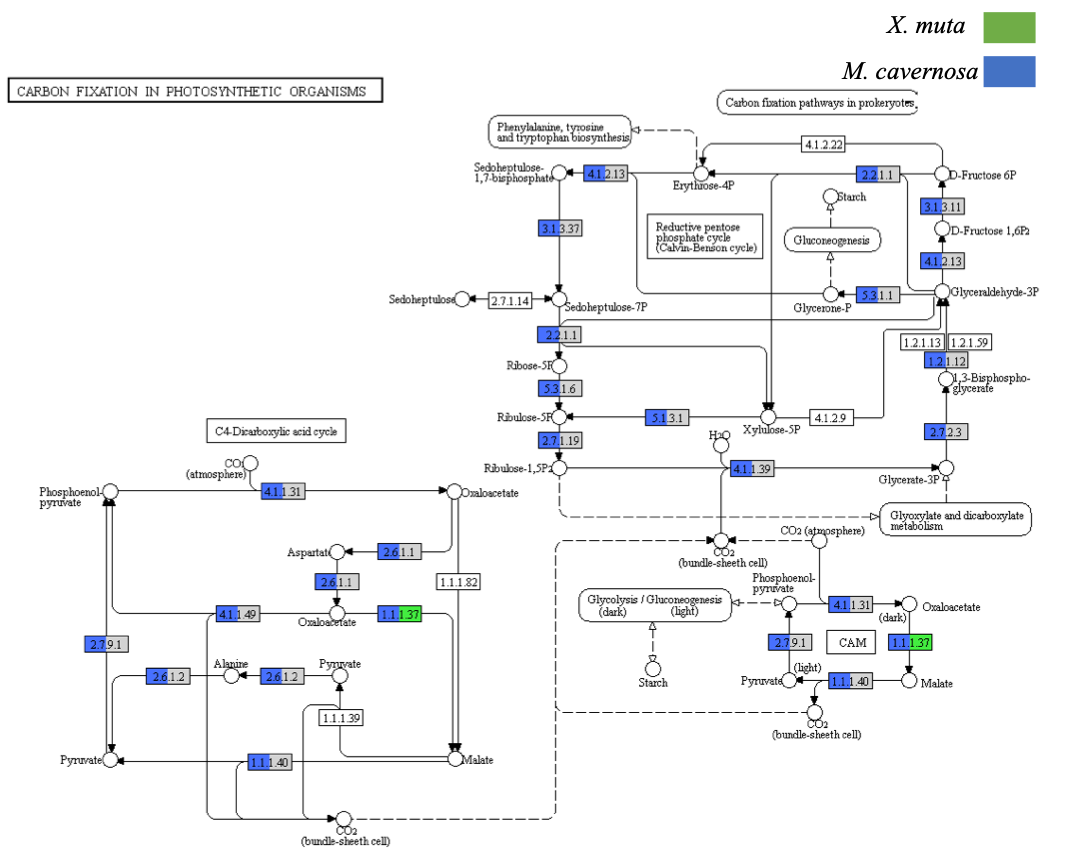


Figure S5. Pathways for carbon fixation in photosynthetic organisms in the sponge and coral microbiomes. Transcripts that mapped to carbon metabolism KEGG pathway^25^ are shown here with color representing the presence of one or more transcripts (green = sponge (*X. muta*), blue = coral (*M. cavernosa*)). Gray color indicates no transcripts were mapped for one of the hosts, no color indicates no transcripts mapped for either host. Visualized with Pathview^28^.


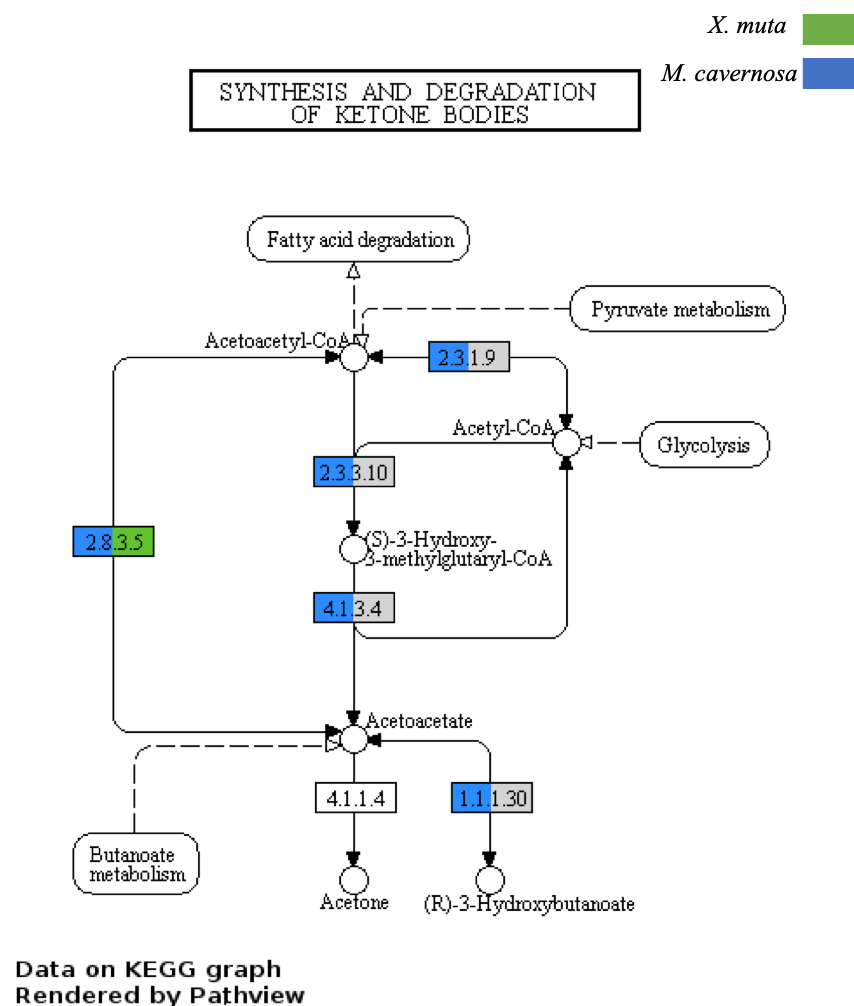


Figure S6. Ketone body metabolism in the sponge and coral microbiomes. Transcripts that mapped to ketone metabolism KEGG pathway^25^ are shown here with color representing the presence of one or more transcripts (green = sponge (*X. muta*), blue = coral (*M. cavernosa*)). Gray color indicates no transcripts were mapped for one of the hosts, no color indicates no transcripts mapped for either host. Visualized with Pathview^28^.

Table S1. Distance values of FTU (Tax4Fun^18^) and NSTI (PICRUSt^17^) from metagenomic predictions based on 16S rDNA amplicon reads for all sponge (*X. muta*) and coral (*M. cavernosa*) samples by color morph (McavOr, McavBr). Sponge samples were collected from different locations which are identified as FL (Florida Keys), LC (Little Cayman Island), LSI (Lee Stocking Island, Bahamas), while corals are from LSI only.

| **Sample** | **FTU** | **NSTI** |
| --- | --- | --- |
| XmutFL1 | 0.65 | 0.30 |
| XmutFL2 | 0.61 | 0.30 |
| XmutFL3 | 0.63 | 0.32 |
| XmutLC1 | 0.47 | 0.11 |
| XmutLC2 | 0.45 | 0.15 |
| XmutLC3 | 0.60 | 0.28 |
| XmutLSI1 | 0.62 | 0.29 |
| XmutLSI2 | 0.55 | 0.26 |
| XmutLSI3 | 0.63 | 0.29 |
| McavBr1 LSI | 0.81 | 0.29 |
| McavBr2 LSI | 0.38 | 0.23 |
| McavBr3 LSI | 0.71 | 0.21 |
| McavOr1 LSI | 0.60 | 0.24 |
| McavOr2 LSI | 0.66 | 0.23 |
| McavOr3 LSI | 0.53 | 0.21 |

Table S2. Top 25 metabolic pathways of coral and sponge microbiomes based on 16S rRNA gene predictions (PICRUSt^17^ and Tax4Fun^18^). The relative abundance of the pathway is shown for each pathway and host. Bolded pathways are in common.

| **Program** | **Sponge (*X. muta*)** | **Average** | **Coral (*M. cavernosa*)** | **Average** |
| --- | --- | --- | --- | --- |
| Picrust | **ko00290: Valine, leucine and isoleucine biosynthesis** | 0.84 | **ko00290: Valine, leucine and isoleucine biosynthesis** | 0.86 |
|  | **ko00471: D-Glutamine and D-glutamate metabolism** | 0.83 | ko04112: Cell cycle - Caulobacter | 0.86 |
|  | **ko02040: Flagellar assembly** | 0.83 | **ko00471: D-Glutamine and D-glutamate metabolism** | 0.83 |
|  | **ko00195: Photosynthesis** | 0.83 | **ko02040: Flagellar assembly** | 0.83 |
|  | **ko00450: Selenocompound metabolism** | 0.82 | **ko00195: Photosynthesis** | 0.82 |
|  | **ko04122: Sulfur relay system** | 0.80 | ko02030: Bacterial chemotaxis | 0.81 |
|  | **ko00720: Carbon fixation pathways in prokaryotes** | 0.79 | **ko00450: Selenocompound metabolism** | 0.78 |
|  | **ko02030: Bacterial chemotaxis** | 0.77 | **ko04122: Sulfur relay system** | 0.77 |
|  | **ko00260: Glycine, serine and threonine metabolism** | 0.77 | **ko00730: Thiamine metabolism** | 0.75 |
|  | **ko00785: Lipoic acid metabolism** | 0.75 | **ko00785: Lipoic acid metabolism** | 0.75 |
|  | **ko00620: Pyruvate metabolism** | 0.75 | **ko00720: Carbon fixation pathways in prokaryotes** | 0.74 |
|  | **ko00020: Citrate cycle (TCA cycle)** | 0.73 | **ko00260: Glycine, serine and threonine metabolism** | 0.73 |
|  | **ko00030: Pentose phosphate pathway** | 0.72 | **ko00030: Pentose phosphate pathway** | 0.72 |
|  | **ko00730: Thiamine metabolism** | 0.71 | **ko00340: Histidine metabolism** | 0.72 |
|  | **ko00340: Histidine metabolism** | 0.71 | **ko00710: Carbon fixation in photosynthetic organisms** | 0.69 |
|  | **ko00670: One carbon pool by folate** | 0.70 | ko00072: Synthesis and degradation of ketone bodies | 0.69 |
|  | **ko00780: Biotin metabolism** | 0.70 | **ko00620: Pyruvate metabolism** | 0.67 |
|  | **ko04112: Cell cycle - Caulobacter** | 0.70 | **ko00670: One carbon pool by folate** | 0.65 |
|  | **ko00710: Carbon fixation in photosynthetic organisms** | 0.69 | ko00760: Nicotinate and nicotinamide metabolism | 0.65 |
|  | ko00650: Butanoate metabolism | 0.68 | **ko00020: Citrate cycle (TCA cycle)** | 0.65 |
|  | ko00540: Lipopolysaccharide biosynthesis | 0.68 | **ko00780: Biotin metabolism** | 0.63 |
|  | ko00633: Nitrotoluene degradation | 0.68 | **ko00250: Alanine, aspartate and glutamate metabolism** | 0.63 |
|  | **ko00250: Alanine, aspartate and glutamate metabolism** | 0.66 | ko00630: Glyoxylate and dicarboxylate metabolism | 0.63 |
|  | ko00900: Terpenoid backbone biosynthesis | 0.66 | ko00770: Pantothenate and CoA biosynthesis | 0.63 |
|  | ko00640: Propanoate metabolism | 0.65 | ko00660: C5-Branched dibasic acid metabolism | 0.63 |

Table S2. Continued

| **Program** | **Sponge (*X. muta*)** | **Average** | **Coral (*M. cavernosa*)** | **Average** |
| --- | --- | --- | --- | --- |
| Tax4Fun | **ko00290: Valine, leucine and isoleucine biosynthesis** | 0.88 | **ko00290: Valine, leucine and isoleucine biosynthesis** | 0.86 |
|  | **ko00471: D-Glutamine and D-glutamate metabolism** | 0.83 | **ko00471: D-Glutamine and D-glutamate metabolism** | 0.83 |
|  | **ko00473: D-Alanine metabolism** | 0.80 | **ko00473: D-Alanine metabolism** | 0.80 |
|  | **ko00450: Selenocompound metabolism** | 0.79 | **ko00281: Geraniol degradation** | 0.77 |
|  | **ko00785: Lipoic acid metabolism** | 0.75 | **ko02040: Flagellar assembly** | 0.77 |
|  | **ko02040: Flagellar assembly** | 0.75 | **ko00450: Selenocompound metabolism** | 0.76 |
|  | **ko00072: Synthesis and degradation of ketone bodies** | 0.74 | **ko04112: Cell cycle - Caulobacter** | 0.76 |
|  | **ko00030: Pentose phosphate pathway** | 0.72 | **ko00785: Lipoic acid metabolism** | 0.75 |
|  | **ko00710: Carbon fixation in photosynthetic organisms** | 0.71 | **ko00030: Pentose phosphate pathway** | 0.72 |
|  | **ko02030: Bacterial chemotaxis** | 0.70 | **ko00710: Carbon fixation in photosynthetic organisms** | 0.71 |
|  | **ko00260: Glycine, serine and threonine metabolism** | 0.68 | **ko02030: Bacterial chemotaxis** | 0.69 |
|  | **ko00900: Terpenoid backbone biosynthesis** | 0.67 | **ko00072: Synthesis and degradation of ketone bodies** | 0.69 |
|  | **ko00750: Vitamin B6 metabolism** | 0.67 | **ko00930: Caprolactam degradation** | 0.68 |
|  | **ko04112: Cell cycle - Caulobacter** | 0.67 | **ko00260: Glycine, serine and threonine metabolism** | 0.68 |
|  | **ko00340: Histidine metabolism** | 0.66 | **ko00625: Chloroalkane and chloroalkene degradation** | 0.65 |
|  | **ko00620: Pyruvate metabolism** | 0.65 | **ko00340: Histidine metabolism** | 0.65 |
|  | **ko00281: Geraniol degradation** | 0.65 | **ko00770: Pantothenate and CoA biosynthesis** | 0.65 |
|  | **ko00625: Chloroalkane and chloroalkene degradation** | 0.65 | **ko00900: Terpenoid backbone biosynthesis** | 0.65 |
|  | ko00791: Atrazine degradation | 0.64 | **ko00620: Pyruvate metabolism** | 0.64 |
|  | **ko00250: Alanine, aspartate and glutamate metabolism** | 0.63 | ko00250: Alanine, aspartate and glutamate metabolism | 0.63 |
|  | **ko00930: Caprolactam degradation** | 0.63 | ko00750: Vitamin B6 metabolism | 0.62 |
|  | ko04122: Sulfur relay system | 0.62 | ko00630: Glyoxylate and dicarboxylate metabolism | 0.60 |
|  | ko00720: Carbon fixation pathways in prokaryotes | 0.62 | ko00540: Lipopolysaccharide biosynthesis | 0.60 |
|  | **ko00770: Pantothenate and CoA biosynthesis** | 0.62 | ko00780: Biotin metabolism | 0.60 |
|  | ko00860: Porphyrin and chlorophyll metabolism | 0.62 | ko00660: C5-Branched dibasic acid metabolism | 0.60 |

Table S3. List of KEGG Ortlogy^25^ pathways that were unique to each analysis method (HUMAnN^26^ analysis following PICRUST^17^ prediction or metatranscriptome). The number and percentage (out of total pathways in both methods) of shared pathways are also shown.

| **Host species** | **Picrust prediction only** | **Metatranscriptome only** | **Number of shared pathways** |
| --- | --- | --- | --- |
| Coral | ko00062: Fatty acid elongation in mitochondria | ko05130: Pathogenic Escherichia coli infection | 130 (80%) |
|  | ko00100: Steroid biosynthesis | ko05140: Leishmaniasis |  |
|  | ko00253: Tetracycline biosynthesis | ko05200: Pathways in cancer |  |
|  | ko00312: beta-Lactam resistance | ko05222: Small cell lung cancer |  |
|  | ko00363: Bisphenol degradation |  |  |
|  | ko00510: N-Glycan biosynthesis |  |  |
|  | ko00513: Various types of N-glycan biosynthesis |  |  |
|  | ko00523: Polyketide sugar unit biosynthesis |  |  |
|  | ko00565: Ether lipid metabolism |  |  |
|  | ko00592: alpha-Linolenic acid metabolism |  |  |
|  | ko00621: Dioxin degradation |  |  |
|  | ko00622: Xylene degradation |  |  |
|  | ko00642: Ethylbenzene degradation |  |  |
|  | ko00791: Atrazine degradation |  |  |
|  | ko00901: Indole alkaloid biosynthesis |  |  |
|  | ko00940: Phenylpropanoid biosynthesis |  |  |
|  | ko00943: Isoflavonoid biosynthesis |  |  |
|  | ko03015: mRNA surveillance pathway |  |  |
|  | ko03022: Basal transcription factors |  |  |
|  | ko04115: p53 signaling pathway |  |  |
|  | ko04621: NOD-like receptor signaling pathway |  |  |
|  | ko04622: RIG-I-like receptor signaling pathway |  |  |
|  | ko05010: Alzheimer's disease |  |  |
|  | ko05012: Parkinson's disease |  |  |
|  | ko05110: Vibrio cholerae infection |  |  |
|  | ko05111: Vibrio cholerae pathogenic cycle |  |  |
|  | ko05143: African trypanosomiasis |  |  |
|  | ko05145: Toxoplasmosis |  |  |
|  | ko05146: Amoebiasis |  |  |
|  | ko05150: Staphylococcus aureus infection |  |  |

Table S3. Continued

| **Host species** | **Picrust prediction only** | **Metatranscriptome only** | **Number of shared pathways** |
| --- | --- | --- | --- |
| Sponge | ko00062: Fatty acid elongation in mitochondria | ko04260: Cardiac muscle contraction | 129 (80%) |
|  | ko00253: Tetracycline biosynthesis | ko05222: Small cell lung cancer |  |
|  | ko00312: beta-Lactam resistance | ko05322: Systemic lupus erythematosus |  |
|  | ko00364: Fluorobenzoate degradation |  |  |
|  | ko00523: Polyketide sugar unit biosynthesis |  |  |
|  | ko00565: Ether lipid metabolism |  |  |
|  | ko00591: Linoleic acid metabolism |  |  |
|  | ko00592: alpha-Linolenic acid metabolism |  |  |
|  | ko00621: Dioxin degradation |  |  |
|  | ko00622: Xylene degradation |  |  |
|  | ko00642: Ethylbenzene degradation |  |  |
|  | ko00643: Styrene degradation |  |  |
|  | ko00830: Retinol metabolism |  |  |
|  | ko00901: Indole alkaloid biosynthesis |  |  |
|  | ko00943: Isoflavonoid biosynthesis |  |  |
|  | ko01056: Biosynthesis of type II polyketide backbone |  |  |
|  | ko03015: mRNA surveillance pathway |  |  |
|  | ko03320: PPAR signaling pathway |  |  |
|  | ko04113: Meiosis - yeast |  |  |
|  | ko04115: p53 signaling pathway |  |  |
|  | ko04210: Apoptosis |  |  |
|  | ko04621: NOD-like receptor signaling pathway |  |  |
|  | ko04622: RIG-I-like receptor signaling pathway |  |  |
|  | ko04962: Vasopressin-regulated water reabsorption |  |  |
|  | ko05010: Alzheimer's disease |  |  |
|  | ko05012: Parkinson's disease |  |  |
|  | ko05145: Toxoplasmosis |  |  |
|  | ko05146: Amoebiasis |  |  |
|  | ko05150: Staphylococcus aureus infection |  |  |

Table S4. List of KEGG Orthology^25^ pathways that were unique to each analysis method (HUMAnN^26^ analysis following Tax4Fun^18^ prediction or metatranscriptome). The number and percentage (out of total pathways in both methods) of shared pathways are also shown.

| **Host species** | **Tax4Fun prediction only** | **Metatranscriptome only** | **Number of shared pathways** |
| --- | --- | --- | --- |
| Coral | ko00100: Steroid biosynthesis | ko00590: Arachidonic acid metabolism | 130 (80%) |
|  | ko00253: Tetracycline biosynthesis | ko03320: PPAR signaling pathway |  |
|  | ko00312: beta-Lactam resistance | ko05140: Leishmaniasis |  |
|  | ko00331: Clavulanic acid biosynthesis | ko05222: Small cell lung cancer |  |
|  | ko00363: Bisphenol degradation |  |  |
|  | ko00510: N-Glycan biosynthesis |  |  |
|  | ko00522: Biosynthesis of 12-, 14- and 16-membered macrolides |  |  |
|  | ko00532: Glycosaminoglycan biosynthesis - chondroitin sulfate |  |  |
|  | ko00563: Glycosylphosphatidylinositol(GPI)-anchor biosynthesis |  |  |
|  | ko00565: Ether lipid metabolism |  |  |
|  | ko00621: Dioxin degradation |  |  |
|  | ko00622: Xylene degradation |  |  |
|  | ko00642: Ethylbenzene degradation |  |  |
|  | ko00791: Atrazine degradation |  |  |
|  | ko00901: Indole alkaloid biosynthesis |  |  |
|  | ko00909: Sesquiterpenoid biosynthesis |  |  |
|  | ko00943: Isoflavonoid biosynthesis |  |  |
|  | ko01056: Biosynthesis of type II polyketide backbone |  |  |
|  | ko03015: mRNA surveillance pathway |  |  |
|  | ko03022: Basal transcription factors |  |  |
|  | ko04621: NOD-like receptor signaling pathway |  |  |
|  | ko04962: Vasopressin-regulated water reabsorption |  |  |
|  | ko05010: Alzheimer's disease |  |  |
|  | ko05110: Vibrio cholerae infection |  |  |
|  | ko05111: Vibrio cholerae pathogenic cycle |  |  |
|  | ko05131: Shigellosis |  |  |
|  | ko05143: African trypanosomiasis |  |  |
|  | ko05146: Amoebiasis |  |  |
|  | ko05150: Staphylococcus aureus infection |  |  |

Table S4. Continued

| **Host species** | **Tax4Fun prediction only** | **Metatranscriptome only** | **Number of shared pathways** |
| --- | --- | --- | --- |
| Sponge | ko00253: Tetracycline biosynthesis | ko00590: Arachidonic acid metabolism | 128 (80%) |
|  | ko00312: beta-Lactam resistance | ko00940: Phenylpropanoid biosynthesis |  |
|  | ko00364: Fluorobenzoate degradation | ko04260: Cardiac muscle contraction |  |
|  | ko00522: Biosynthesis of 12-, 14- and 16-membered macrolides | ko05222: Small cell lung cancer |  |
|  | ko00532: Glycosaminoglycan biosynthesis - chondroitin sulfate |  |  |
|  | ko00563: Glycosylphosphatidylinositol(GPI)-anchor biosynthesis |  |  |
|  | ko00565: Ether lipid metabolism |  |  |
|  | ko00591: Linoleic acid metabolism |  |  |
|  | ko00621: Dioxin degradation |  |  |
|  | ko00622: Xylene degradation |  |  |
|  | ko00642: Ethylbenzene degradation |  |  |
|  | ko00643: Styrene degradation |  |  |
|  | ko00830: Retinol metabolism |  |  |
|  | ko00901: Indole alkaloid biosynthesis |  |  |
|  | ko00909: Sesquiterpenoid biosynthesis |  |  |
|  | ko00943: Isoflavonoid biosynthesis |  |  |
|  | ko01056: Biosynthesis of type II polyketide backbone |  |  |
|  | ko03015: mRNA surveillance pathway |  |  |
|  | ko04113: Meiosis - yeast |  |  |
|  | ko04210: Apoptosis |  |  |
|  | ko04621: NOD-like receptor signaling pathway |  |  |
|  | ko04962: Vasopressin-regulated water reabsorption |  |  |
|  | ko05010: Alzheimer's disease |  |  |
|  | ko05100: Bacterial invasion of epithelial cells |  |  |
|  | ko05110: Vibrio cholerae infection |  |  |
|  | ko05130: Pathogenic Escherichia coli infection |  |  |
|  | ko05131: Shigellosis |  |  |
|  | ko05146: Amoebiasis |  |  |
|  | ko05150: Staphylococcus aureus infection |  |  |
|  | ko05200: Pathways in cancer |  |  |
